# Screening, Synthesis, and Characterization of a More Rapidly Dissolving Celecoxib Crystal Form

**DOI:** 10.1021/acsomega.4c03188

**Published:** 2024-06-27

**Authors:** Aaron O’Sullivan, Enrico Spoletti, Steven A. Ross, Matteo Lusi, Dennis Douroumis, Kevin M. Ryan, Luis Padrela

**Affiliations:** † SSPC Research Centre, University of Limerick, Limerick V94 T9PX, Ireland; ‡ Department of Chemical Sciences, Bernal Institute, University of Limerick, Limerick V94 T9PX, Ireland; § CIPERinitio Centre for Innovation and Process Engineering Research, University of Greenwich, Chatham, Maritime Kent ME4 4TB, U.K.; ∥ Custom Pharma Services, Brighton and Hove, East Sussex BN3 3LW, U.K.

## Abstract

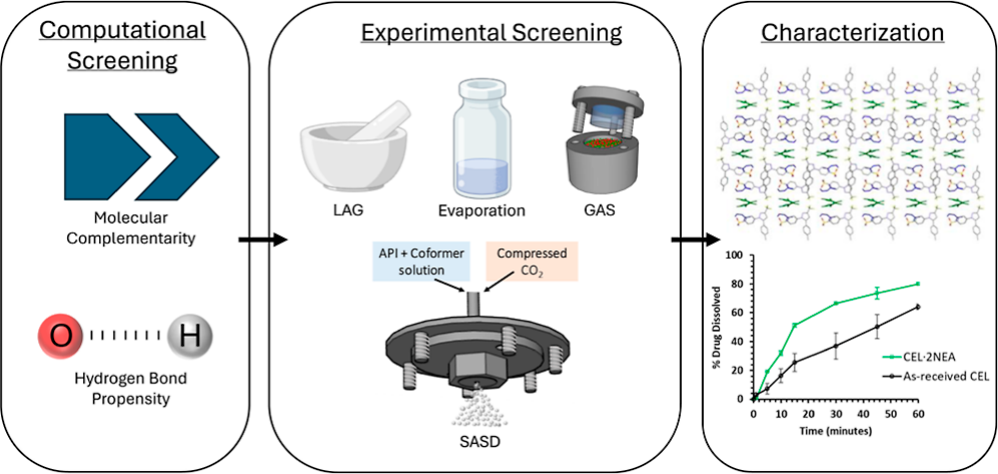

The prevalence of poor solubility in active pharmaceutical ingredients (APIs) such as celecoxib (CEL) is a major bottleneck in the pharmaceutical industry, leading to a low concentration gradient, poor passive diffusion, and in vivo failure. This study presents the synthesis and characterization of a new cocrystal of the API CEL. CEL is a nonsteroidal anti-inflammatory drug used for the treatment of osteoarthritis and rheumatoid arthritis. Computational screening was completed for CEL against a large library of generally recognized as safe (GRAS) coformers, based on molecular complementarity and hydrogen bond propensity (HBP). The generated list of 17 coformers with a likelihood for cocrystallization with CEL were experimentally screened using four techniques: liquid-assisted grinding (LAG), solvent evaporation (SE), gas antisolvent crystallization (GAS), and supercritical enhanced atomization (SEA). One new crystalline form was isolated, employing the liquid coformer *N*-ethylacetamide (NEA). This novel form, celecoxib-di-*N*-ethylacetamide (CEL·2NEA), was characterized by a variety of different techniques. The crystal structure was determined through single-crystal X-ray diffraction. Both NEA molecules are evolved from the crystal structure at a desolvation temperature of approximately 65 °C. The CEL·2NEA cocrystal exhibited a dissolution rate, with more than a twofold improvement in comparison to as-received CEL after only 15 min.

## Introduction

The generation of multicomponent solid forms of active pharmaceutical ingredients (APIs) such as salts,^1^ cocrystals,^2^ hydrates,^3^ and solvates^4^ to improve the physicochemical properties of pharmaceutical compounds has been well established, enhancing the solubility and/or dissolution rate of many APIs.^5,6^ While each of these strategies has proven successful for a range of APIs, it is not a “one size fits all” arrangement. Salts require the presence of ionizable groups on reactant molecules, while cocrystals require molecular complementarity (MC) and a relatively high degree of hydrogen bond propensity (HBP),^7^ requirements that are not met by all API and coformer pairs. As such, appropriate screening protocols need to be put in place to determine the likelihood of producing such multicomponent crystal forms to limit the need for excessively large experimental screening studies. It is noted that there is some debate in the literature as to the distinction between solvates and cocrystals regarding the use of coformers which are liquid at room temperature.^8^ Solvates are regarded as subclasses of cocrystals in this instance, as has been noted previously.^9^

Presently, while computational screening methods are becoming more apparent, experimental screening of a large library of coformers with a single API is still the most popular method for cocrystal discovery in the literature.^10,11^ Experimental screening studies primarily employ a large list of coformers and perform rapid benchtop testing, employing methods such as slurrying, solvent evaporation (SE), and various mechanochemistry methods.^10^ While these methods have proved successful in the past, they can be time-consuming and costly to develop such a large library of common coformers. Regarding knowledge-based approaches, Aakeröy et al. sought to design cocrystals comprised of three components by employing p*K*_a_ values to identify hydrogen-bond donors.^12^ Makadia et al.^7^ employed computational methods such as MC, HBP, and hydrogen-bond energy to screen a list of coformers to determine the likelihood of cocrystal formation, while Musumeci et al.^13^ investigated electrostatic surface potentials to identify pairs of hydrogen-bond donor and acceptor sites as a virtual cocrystal screening tactic. Cappuccino et al.^14^ determined that complementarity screening based on geometrical and energetic factors lead to a 75% reduction in experiments carried out. However, due to the strong dependence on the chosen molecular conformation, some new forms may be missed. Many methods that employ supercritical fluids such as the supercritical enhanced atomization(SEA) method and the gas antisolvent crystallization (GAS) method can allow for rapid screening of coformer candidates also but have not been compared in this regard to more conventional screening methods such as liquid-assisted grinding (LAG) and solvent evaporation (SE).

**Figure 1:**
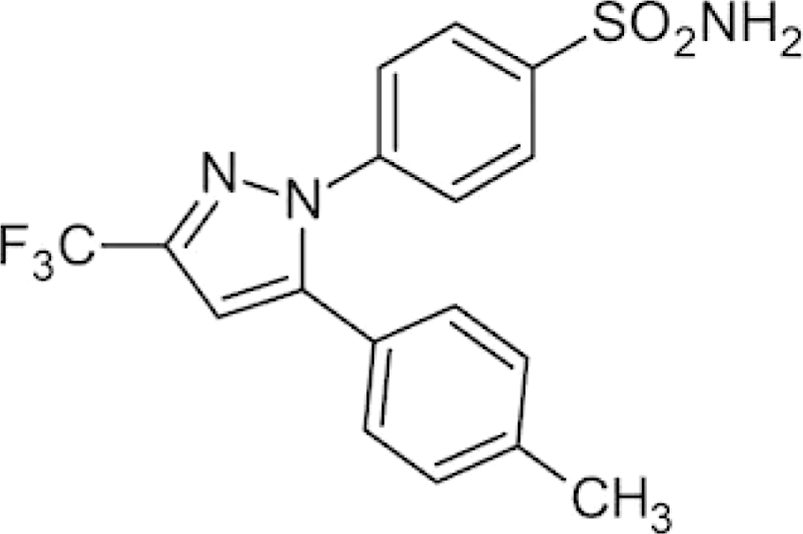
Chemical structure of celecoxib (CEL).

Celecoxib (CEL) is a small molecule which exhibits poor bioavailability due to its poor aqueous solubility (∼3.2 ± 0.1 μg/mL^15^). For this reason, it is classified as a Biopharmaceutical Classification System (BCS) class II drug. It is a nonsteroidal anti-inflammatory drug (NSAID) for the treatment of pain in conditions such as rheumatism and osteoarthritis, with its chemical structure presented in Figure 1. Several methods have been employed in the literature to enhance the solubility of this drug such as β-cyclodextrin inclusion complexes which demonstrated a dissolution rate ∼23 times greater than the pure drug,^16^ and solid dispersions which led to an increase in the dissolution rate up to 200%.^15,17^ Many solid crystalline forms of this API have also been reported in the literature, including four polymorphic forms with varying stabilities (III > I > II > IV),^18^ 12 cocrystals,^19−25^ 1 trimorphic cocrystal,^24^ 6 solvates,^26^ a sodium salt, a sodium salt hydrate, and several hydrated sodium salt solvates.^27^ In 2021, the CEL–tramadol HCl cocrystal was approved by the FDA for the treatment of acute pain in adults, employing both APIs as a synergistic approach.^28^

The study presented herein employs computational and experimental coformer screening with the aim of producing a new multicomponent crystalline form with CEL. A library of 103 coformers were screened against CEL using two prominent predictive tools: MC and HBP. The reduced list of coformers with a likelihood of hydrogen bond/synthon formation were experimentally screened using two benchtop techniques, LAG and SE, and two supercritical CO_2_-based methods, GAS and SEA to compare these methods in terms of their versatility. Powder X-ray diffraction (PXRD) was the primary method used to elucidate the presence of a new solid form of CEL. Differential scanning calorimetry (DSC), scanning electron microscopy (SEM), Fourier transform infrared (FTIR) spectroscopy, variable temperature PXRD (VT-PXRD), and thermogravimetric analysis (TGA) were employed to characterize the new multicomponent solid form and determine its physicochemical properties, while single-crystal X-ray diffraction (SC-XRD) was used to determine the crystal structure. Dissolution studies were performed under sink conditions to assess the bioavailability enhancement that the reported solid form is capable of.

## Materials and Methods

### Materials

HPLC-grade methanol (>99.9% pure) and *N*-ethylacetamide (NEA) (99% pure) were purchased from Sigma-Aldrich without any further purification. Carbon dioxide (liquid withdrawal) was purchased from BOC gases (>99.98% pure). CEL (99.7% pure), 3-methylpyridine (99% pure), pyrazine (99% pure), l-pyroglutamic acid (99% pure), riboflavin (99% pure), alitame (>98% pure), 4-aminobenzoic acid (99% pure), l-glutathione (98% pure), *trans*-cinnamic acid (99% pure), lactose (98% pure), l-epicatechin (90% pure), phthalimide (99%), thymidine (98% pure), biotin (99% pure), l-fucose (95% pure), and 2-amino-5-methylbenzoic acid (98% pure) were purchased from Baoji GuoKang Bio-Technology Co., Ltd.

### Computational Coformer Screening

Ten different conformations for CEL were generated and screened against a previously generated library of 103 generally recognized as safe (GRAS) coformers. Briefly, these coformers were screened on the basis of MC, and those which failed this computational screening were not considered for the experimental screening. API:coformer combinations which led to at least one positive “hit” were further analyzed on the basis of HBP. The coformers in the final results of this screening were first ranked based on their MC, and secondary to this, their likelihood of supramolecular (hetero)synthon formation was ranked based on HBP.

#### Coformer Generation

During this initial stage of screening, 10 different conformations of CEL were generated and screened against a library of coformers. The purpose of this initial screening was to ensure that the target coformers have a degree of flexibility to form cocrystals with a variety of molecular conformations. Coformers with compatibility to a limited number of API conformations indicate rigidity and are unlikely to form cocrystals in practice. Only molecules with 10 hits were selected for further analysis, as listed in Table 1.

**Table 1:** Computational Coformer Screening Results, Generated through Coformer Generation, MC, and HBP^a^

coformer generation					molecular complementarity			HBP	
rank	coformer	number of hits (pass > 10)	*M*/*L* axis ratio δ (pass < 0.31)	*S* axis (Å) δ (pass < 3.23)	*S*/*L* axis ratio δ (pass < 0.275)	dipole moment magnitude (Debye) δ (pass < 5.94)	fraction of nitrogen and oxygen δ (pass < 0.294)	overall complementarity screen (pass > 9 hits)	MCS
1	3-methylpyridine	10	0.091	1.823	0.086	2.981	0.049	pass	0.18
2	pyrazine	10	0.001	2.579	0.105	4.418	0.141	pass	0.18
3	l-pyroglutamic acid	10	0.185	0.748	0.162	2.831	0.252	pass	0.13
4	riboflavin	10	0.072	1.005	0.082	0.44	0.178	pass	0.11
5	alitame	10	0.248	1.999	0.19	2.437	0.126	pass	0.08
6	4-aminobenzoic acid	10	0.234	2.59	0.079	3.568	0.3	pass	0.07
7	l-glutathione	10	0.066	0.865	0.058	3.772	0.258	pass	0.06
8	*N*-ethylacetamide	10	0.22	1.821	0.058	2.762	0.141	pass	0.05
9	*trans*-cinnamic acid	10	0.287	2.471	0.11	3.772	0.01	pass	0.03
10	valerolactam	10	0.056	0.855	0.229	2.39	0.093	pass	0.06
11	lactose	10	0.218	0.602	0.095	0.137	0.286	pass	0.03
12	l-epicatechin	10	0.069	1.765	0.125	1.256	0.128	pass	0.03
13	thymidine	10	0.205	1.552	0.226	0.003	0.219	pass	0.03
14	biotin	10	0.246	0.334	0.051	2.78	0.12	pass	0.01
15	phthalimide	10	0.05	1.046	0.138	1.543	0.141	pass	0.01
16	l-fucose	10	0	0.544	0.198	2.421	0.262	pass	0
17	2-amino-5-methylbenzoic acid	10	0.137	1.821	0.012	3.057	0.08	pass	0.02

a HBP: hydrogen bonding propensity, *S*: small axis, *M*: medium axis, *L*: large axis, and MCS: multicomponent score.

#### Molecular Complementarity

Molecular complementary is a further predictive tool employed to narrow the list of coformers to those more likely to form cocrystals with a given API. This is based upon the “principle of close packing” which represents two main descriptors, polarity and molecular shape, which are strong indicators for likely cocrystal formation.^7,29^ Specifically, two polarity descriptors (nitrogen/oxygen fraction and dipole moment) and three shape descriptors (*S* axis, *S*/*L* axis, and *M*/*L* axis) were investigated. Polarity descriptors were investigated as cocrystals tend to form between reactants with similar polarities, with the dipole moment displaying the strongest correlation. Shape descriptors are investigated, as cocrystallization is also more likely to occur between reactants with similar geometries. The van der Waals volume of a molecule is enclosed in a box with three axes, long (*L*), medium (*M*), and short (*S*). While these three descriptors detail the molecular size, their ratios provide an insight into the molecular shape.^29^

Each of these five descriptors will display a pass or fail based on the above stated criteria. As the coformers will be screened against the 10 different API conformations, those which pass in all 5 descriptors for more than 90% of the API confirmations will be regarded as “passing” MC and will proceed to HBP screening.

#### Hydrogen Bond Propensity

HBP is a final cocrystal predictive tool employed here that investigates the probability of a specific hydrogen bond forming between specified functional groups on the API and coformer. This propensity prediction is more useful than hydrogen-bond frequencies, as it takes into account factors such as steric hindrance, competition, and aromaticity.^30−32^ An identifying motif in cocrystals is the ability of the two constituents to form supramolecular heterosynthons between one another. Synthons are the basic structural units within supermolecules, which form through noncovalent bonding, consisting of molecular fragments and the supramolecular associations between them. Synthons can be either homosynthons, composed of self-complementary functional groups within the same molecule, or heterosynthons, composed of different but complementary functional groups. This method first calculates the strongest homomeric bond, API–API or coformer–coformer, and the strongest heteromeric bond (API–coformer) and subtracts both to get the difference, which is termed the multicomponent score (MCS).^32^ API–coformer pairs with an MCS greater than 0 indicate that the strongest potential interaction will result in the formation of a supramolecular heterosynthon between the two constituents, indicating the likelihood of cocrystal formation. Conversely, negative values indicate a stronger possibility of API–API interactions or coformer–coformer interactions. The final shortened list of coformers, as summarized in Table 1, are ranked first in the order of complementarity across the 10 different API conformations, MCS, and finally by individual complementarity parameters.

### Experimental Screening Methods

#### Liquid-Assisted Grinding

Liquid assisted grinding (LAG) was performed by using a ceramic pestle and mortar. Samples were ground in a 1:1 molar ratio, using 100 mg of CEL and the coformer mass according to Table 2. Each mixture was ground for 15 min adding methanol in a dropwise fashion. For the two liquid coformers (3-methylpyridine and NEA), methanol was not incorporated, as the coformers themselves act as the liquid, and these samples were left to dry in an oven at 45 °C after grinding. All ground samples were collected and stored in a desiccator.

**Table 2:** Coformer Mass Employed for LAG with 100 mg of CEL in a 1:1 Molar Ratio^a^

sample name	coformer	coformer mass (mg)
**LAG1**	3-methylpyridine	24
**LAG2**	pyrazine	21
**LAG3**	l-pyroglutamic acid	34
**LAG4**	riboflavin	99
**LAG5**	alitame	87
**LAG6**	4-aminobenzoic acid	36
**LAG7**	l-glutathione	81
**LAG8a**	*N*-ethylacetamide	23
**LAG8b**^a^	*N*-ethylacetamide^a^	46^a^
**LAG9**	*trans*-cinnamic acid	39
**LAG10**	valerolactam	N/A
**LAG11**	lactose	90
**LAG12**	l-epicatechin	76
**LAG13**	thymidine	64
**LAG14**	biotin	64
**LAG15**	phthalimide	39
**LAG16**	l-fucose	43
**LAG17**	2-amino-5-methylbenzoic acid	40

a Carried out in a 1:3 molar ratio.

#### Solvent Evaporation

Solvent evaporation (SE) was another “benchtop crystallization method” employed in this work for cocrystallization. This involved the dissolution of API and coformer mixtures in appropriate solvents, as listed in Table 3, provided that the solubility of APIs and coformers in particular solvents is sufficiently high. API and coformer mixtures were dissolved in 5 mL of solvent and left stirring for 2 h at ambient conditions. The solutions were then filtered using a 0.45 μm PTFE filter to remove any undissolved material, and samples were left to evaporate for 24 h at ambient conditions. The collected and dried powders were stored in a desiccator.

**Table 3:** Quantities of CEL and Coformer (1:1 Molar Ratio) along with Associated Solvents Employed for SE^a^

sample reference	CEL concentration (mg/mL)	coformer	coformer concentration (mg/mL)	solvent
**SE1**	50	3-methylpyridine	12	methanol
**SE2**	50	pyrazine	11	methanol
**SE3**	50	l-pyroglutamic acid	17	methanol
**SE4**	20	riboflavin	20	methanol
**SE5**	8	alitame	7	methanol
**SE6**	50	4-aminobenzoic acid	18	methanol
**SE7**	5	l-glutathione	4	ethanol
**SE8**	50	*N*-ethylacetamide	11	methanol
**SE9**	25	*trans*-cinnamic acid	9	methanol
**SE10**^b^	N/A	valerolactam	N/A	N/A
**SE11**^a^	N/A	lactose	N/A	N/A
**SE12**	20	l-epicatechin	15	methanol
**SE13**	10	thymidine	6	methanol
**SE14**^a^	N/A	biotin	N/A	N/A
**SE15**	20	phthalimide	7	methanol
**SE16**	20	l-fucose	7	methanol
**SE17**	20	2-amino-5-methylbenzoic acid	8	methanol

a Could not be run due to solubility constraints.

b Not screened as it is already reported to form a cocrystal with CEL.

#### Gas Antisolvent Crystallization

**Figure 2:**
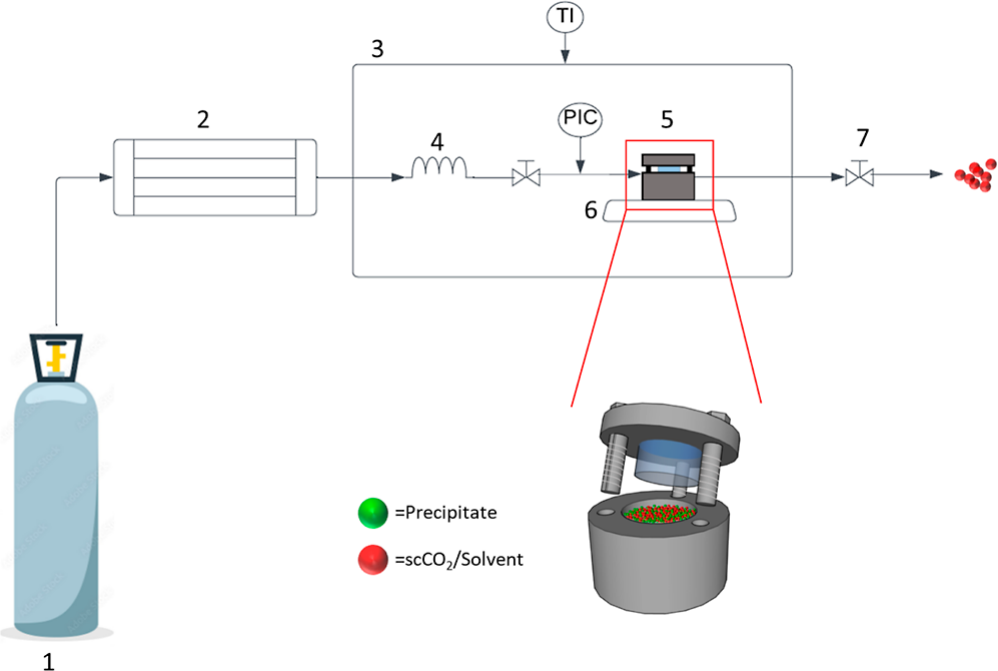
Schematic diagram of the GAS apparatus consisting of (1) CO_2_ cylinder, (2) high-pressure pump, (3) temperature-controlled air chamber, (4) stainless steel storage coil, (5) high-pressure crystallization vessel, (6) magnetic stirrer plate, and (7) exit valve. TI: temperature indicator; PIC: pressure transducer.

Figure 2 represents a schematic of the GAS experimental setup employed for CEL coformer screening for a selected number of coformers, dependent on API and coformer solubilities. The prepared 1 mL solutions (according to concentrations detailed in Table 4 along with a PTFE-coated magnetic stirrer bar were placed in a 10 cm^3^ stainless steel high-pressure vessel (8.83 cm^3^ working volume) (position 5), fitted with a borosilicate window allowing for visual monitoring of the crystallization events. The vessel was sealed and placed in a temperature-controlled air chamber at position 3. CO_2_ from the storage cylinder at position 1 entered the pump (Teledyne ISCO 260D pump) at position 2 where it was cooled to −7 °C and compressed to 120 bar. The CO_2_ then entered a 15 cm^3^ stainless steel storage coil where it remained for 10 min to allow the pressure and temperature to reach the desired values of 50 °C and 120 bar. Temperature and pressure were monitored by T-type thermocouple and a pressure transducer (Omega model PX603), respectively. After 10 min, a valve was opened allowing the CO_2_ into the high-pressure vessel containing the solution at a flow rate of 46 g/min. Once the pressure in the vessel reached 120 bar, the magnetic stirrer was turned on to 600 rpm, facilitating mixing of the supercritical CO_2_ and CEL:coformer solution. After 5 min, the magnetic stirrer bar was switched off and the exit valve at position 7 was opened to flush supercritical CO_2_ through the vessel at a flow rate of approximately 10 g/min for complete removal of solvent. Once all of the CO_2_ (266 mL) had passed through the vessel and the system could be depressurized to atmospheric pressure, the vessel was removed, and the product was collected and stored in a desiccator.

**Table 4:** Quantities of CEL and Coformer (1:1 Molar Ratio) along with Associated Solvents Employed for GAS^a^

sample reference	CEL concentration (mg/mL)	coformer	coformer concentration (mg/mL)	solvent
**GAS1**	50	3-methylpyridine	12	methanol
**GAS2**	50	pyrazine	11	methanol
**GAS3**	50	l-pyroglutamic acid	17	methanol
**GAS4**	20	riboflavin	20	methanol
**GAS5**	8	alitame	7	methanol
**GAS6**	50	4-aminobenzoic acid	18	methanol
**GAS7**	5	l-glutathione	4	ethanol
**GAS8**	50	*N*-ethylacetamide	11	methanol
**GAS9**	25	*trans*-cinnamic acid	9	methanol
**GAS10**^c^	N/A	valerolactam	N/A	N/A
**GAS11**^b^	N/A	lactose	N/A	N/A
**GAS12**	20	l-epicatechin	15	methanol
**GAS13**	10	thymidine	6	methanol
**GAS14**^b^	N/A	biotin	N/A	N/A
**GAS15**	20	phthalimide	7	methanol
**GAS16**	20	l-fucose	7	methanol
**GAS17**	20	2-amino-5-methylbenzoic acid	8	methanol

a Pressure, 120 bar; temperature, 50 °C; and stirring speed, 600 rpm.

b Could not be run due to solubility constraints.

c Not screened as it is already reported to form a cocrystal with CEL.

#### Supercritical Enhanced Atomization (SEA)

**Figure 3:**
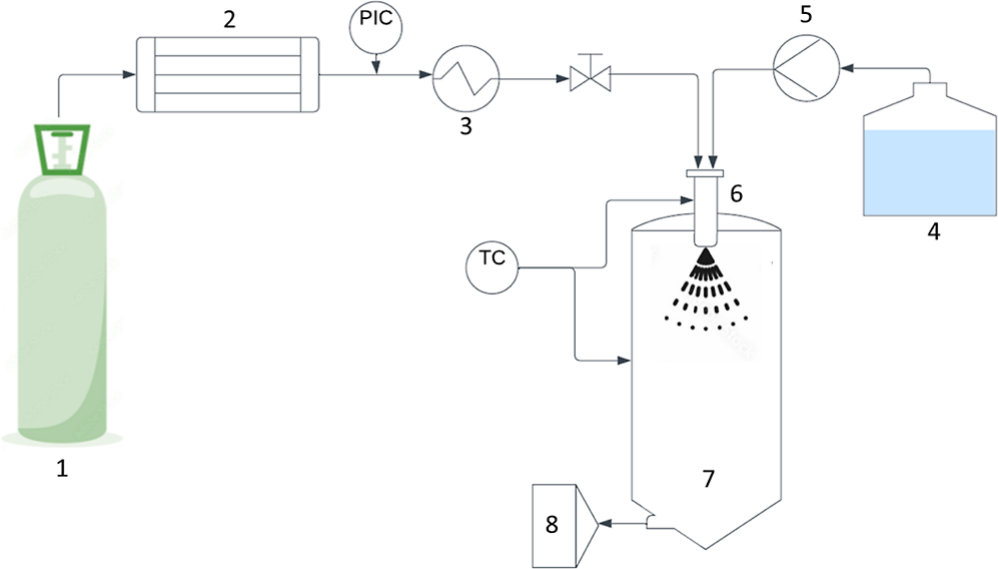
Schematic diagram of the CO_2_-assisted spray-drying apparatus, consisting of (1) CO_2_ liquid withdrawal cylinder, (2) compressor, (3) heat exchanger, (4) solution reservoir, (5) HPLC pump, (6) coaxial nozzle, (7) atomization chamber, and (8) wire mesh (particle collection). TC: temperature controller, PIC: pressure indicator.

Particle production by the SEA process was performed as described elsewhere.^33^ Briefly, similar to the above-mentioned GAS method, the CO_2_ first was chilled to −7 °C before being compressed by the CO_2_ high-pressure pump (P50 Waters) at position 2 of the schematic of Figure 3. During this time, the coaxial nozzle (position 6) and the precipitation chamber (position 7) were heated to the desired temperature of 50 °C. The temperature of the nozzle, which was comprised of five 40 μm pores, was maintained by heating resistors placed in close proximity to the nozzle, while the precipitation chamber was heated by means of a water jacket and an associated heated water bath and pump. The feed solution located at position 4 was prepared according to Table 5 and was pumped to the nozzle at a flow rate of 0.2 mL/min using an Agilent Technologies 1260 Infinity II HPLC pump at position 5. The heated and pressurized CO_2_ and the feed solution meet in the coaxial nozzle (6) where subsequent mixing and atomization occur in the heated drying chamber at atmospheric pressure. The droplets produced at this atomization stage dry by thermal means, leaving behind a suspended powder, which will flow, along with the gaseous CO_2_ and evaporated solvent, to the 10 μm wire mesh filter at position 8 where product separation occurs. The collected, dried powdered samples were collected periodically throughout the process to prevent any excessive build-up on the filter. The final collected product was stored in a desiccator.

**Table 5:** Quantities of CEL and Coformer (1:1 Molar Ratio) along with Associated Solvents Employed for SEA^a^

sample reference	API concentration (mg/mL)	coformer	coformer concentration (mg/mL)	solvent
**SEA1**	50	3-methylpyridine	12	methanol
**SEA2**	50	pyrazine	11	methanol
**SEA3**	50	l-pyroglutamic acid	17	methanol
**SEA4**^b^	20	riboflavin	20	methanol
**SEA5**^c^	8	alitame	7	methanol
**SEA6**	50	4-aminobenzoic acid	18	methanol
**SEA7**^c^	5	l-glutathione	4	ethanol
**SEA8a**	50	*N*-ethylacetamide	11	methanol
**SEA8b**	50	*N*-ethylacetamide	23	methanol
**SEA9**	25	*trans*-cinnamic acid	9	methanol
**SEA10**^d^	N/A	valerolactam	N/A	N/A
**SEA11**^c^	N/A	lactose	N/A	N/A
**SEA12**	20	l-epicatechin	15	methanol
**SEA13**	10	thymidine	6	methanol
**SEA14**^c^	N/A	biotin	N/A	N/A
**SEA15**	20	phthalimide	7	methanol
**SEA16**	20	l-fucose	7	methanol
**SEA17**	20	2-amino-5-methylbenzoic acid	8	methanol

a Temperature, 50 °C; pressure, 120 bar; nozzle, five 40 μm pores.

b Could not be run due to solubility constraints.

c Could not be run due to blocking of the nozzle.

d Not screened as it is already reported to form a cocrystal with CEL. SEA8b was carried out in a 1:2 molar ratio.

### Solid-State Characterization

#### Powder X-ray Diffraction

PXRD in reflection mode was performed using an Empyrean diffractometer (Phillips, PANalytical) with a Cu Kα radiation source (λ = 1.5406 Å) at room temperature. The system was run with a tube current and voltage of 40 mA and 40 kV, respectively, with a scan speed and step size of 0.04 2θ/s and 0.0033°, respectively. An angular range of 5–41° 2θ and a rotational speed of 4 s were also used.

#### Synthesis and Characterization of Single Crystals

For the synthesis of single crystals of the celecoxib-di-*N*-ethylacetamide (CEL·2NEA) cocrystal, 4 mL of NEA was heated to 50 °C and an excess of CEL was dissolved in it. The cloudy solution was then filtered through a 0.2 μm PTFE filter and left to cool at room temperature in an open Petri dish, until single crystals formed. A suitable single crystal was selected and analyzed by SC-XRD. The crystal structure was determined at room temperature on a Bruker D8 QUEST diffractometer equipped with Mo Kα (λ = 0.71073 Å) radiation and a Photon 100 detector. The data were integrated with Bruker SC-XRD software APEX 4. The structure was solved by the intrinsic phasing methods and refined by least-squares methods against *F*_obbs_^2^ using SHELXT^34^ and SHELXL^35^ with the OLEX2^36^ interface. Non-hydrogen atoms were refined anisotropically. Hydrogen atoms were placed in a calculated position. The software Mercury 2022.3.1 was used for graphical representations.^37^

#### Differential Scanning Calorimetry

DSC was performed using 2–5 mg of sample in crimped aluminum pans (30 μL) with a pierced hole in the lid. Measurements were performed in a TA Instrument DSC Q20 using temperature ranges of 40–180 °C and a heating rate of 1 °C/min under a continuous purge of dry nitrogen (flow rate of 50 mL/min).

#### Thermogravimetric Analysis

TGA was carried out in a PerkinElmer TGA 4000 under a heating rate of 10 °C/min with a temperature range of 30–375 °C under a 50 mL/min flux of nitrogen to the sample chamber.

#### Crystal Habit Analysis

The crystal habit of as-received CEL and the new CEL·2NEA cocrystal produced by SEA was assessed by SEM using a Hitachi SU-70 system operating at 5 kV. Samples were adhered by double-sided carbon tape to aluminum stubs and placed in an Emitech k5500x gold sputter coater and coated for 2 min with a plasma current of 20 mA.

The crystal habit of the single crystals of CEL·2NEA was assessed by optical microscopy using an Olympus BX51 polarized light microscope with a small quantity of the sample placed onto a glass slide.

#### FTIR Spectroscopy

FTIR spectroscopy was performed using a Thermo Scientific Nicolet iS50 FT-IR spectrometer with the collecting program OMNIC, in the range of 400–4000 cm^–1^. After background collection, the sample was placed on the crystal and uniformly compacted by the “pushing arm”. A total of 64 scans were performed for each sample measurement.

#### VT-PXRD

Diffractograms at different temperatures were recorded using a PANalytical X’Pert MPD Pro diffractometer equipped with a X’Celerator detector, operating in scanning line detector mode, and an incident beam of Cu Kα radiation (λ = 1.5418 Å) in the 2θ range between 5° and 40° (step size: 0.0167113°; time/step: 29.845 s; soller slit: 0.04 rad; divergence slit: 1/2; 40 mA × 40 kV). Anton Paar TTK 450 stage coupled with the Anton Paar TCU 110 temperature control unit was used to record the variable temperature diffractograms. The powder was loaded on a zero-background sample holder made for the Anton Paar TTK 450 chamber. Measurements were performed under nitrogen stream between 25 and 150 °C, at a 10 °C/min heating rate, and then cooling back to 25 °C.

#### Dissolution Study

The dissolution profiles of as-received CEL and CEL·2NEA (produced by cooling crystallization) were examined in duplicate, in 200 mL of deionized water with 0.25% (w/v) sodium dodecyl sulfate (SDS) at 37 °C, under constant stirring by a magnetic stirrer at 300 rpm. Sink conditions were employed here, with a maximum final CEL concentration of 7 mg/L. Samples were withdrawn at regular intervals (1, 5, 10, 15, 30, 45, and 60 min) and filtered using a 0.2 μm syringe filter. The concentration of dissolved CEL was determined by HPLC analysis using a 1260 Infinity II LC system. HPLC was carried out using a reversed-phase column XBridge C18 Waters (4.6 mm × 150 mm, 3.5 μm). An isocratic mobile phase was used and consisted of 50:50 0.1% TFA in water and 0.1% TFA in acetonitrile, and the UV detector was set to 254 nm. The flow rate was maintained at 0.5 mL/min with a sample injection volume of 10 μL.

## Results and Discussion

### Computational Screening

Of the 103 coformers that were initially included in the computational screening library, 17 achieved a pass in the required criterion regarding MC and HBP, as listed in Table 1. Any MCS greater than 0.10 is said to exhibit a high likelihood of heterosynthon formation, with four coformers exhibiting this: 3-methylpyridine, pyrazine, l-pyroglutamic acid, and riboflavin. Two of these coformers, 3-methylpyridine and pyrazine, also exhibit very similar geometries to CEL, while l-pyroglutamic acid displays a large difference in fractional polar molecules between it and the CEL, which may lead to complications, while riboflavin is considered poorly water-soluble, a further issue in enhancing the bioavailability of CEL.

For MCS values lower than 0.10, heterosynthon formation is still a possibility, with some notable coformers to discuss. 4-Aminobenzoic acid displays a very similar *S*/*L* axis ratio to CEL with similar fractional polar volumes also making it a strong candidate, while NEA displays exceptionally similar geometries to CEL, providing a high likelihood of synthon formation also. This coupled with its high-water solubility makes it an excellent candidate. Another notable result is that of valerolactam, a previously reported cocrystal with CEL.^24^ While this coformer will not be experimentally screened due to this, it is noteworthy that a successful coformer candidate was ranked lower than other coformers here, indicating that while the ranking system employed here has a theoretical basis, exceptions to the rule exist.

### Experimental Screening

**Figure 4:**
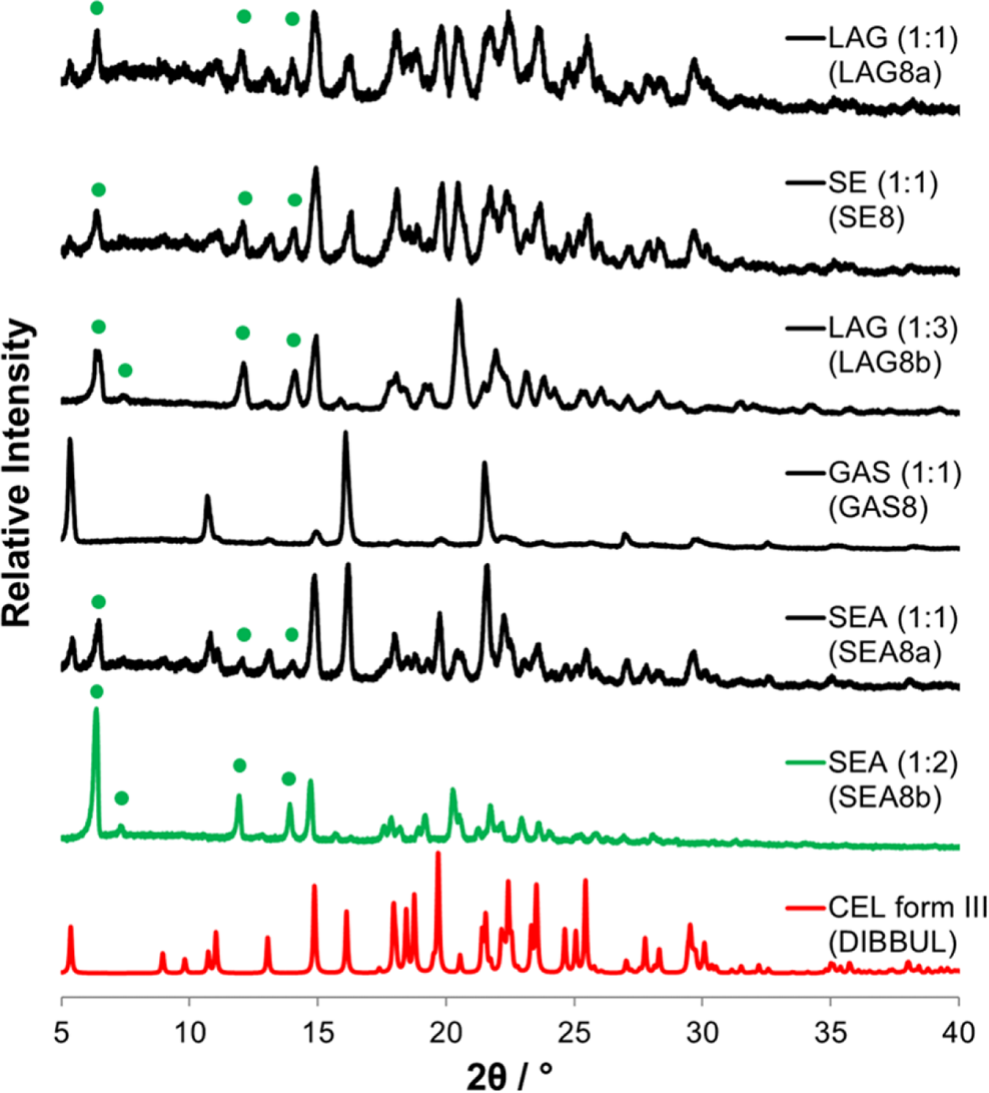
PXRD data for CEL samples coprocessed with NEA by LAG, SE, GAS, and SEA.

The discovery of potential new multicomponent forms of CEL was assessed by PXRD. The presence of additional peaks is implicated in the formation of a new crystalline form worthy of further analysis. The structure of all experimentally tested coformers can be found in Figure S1 in the Supporting Information. Coformers that provide no evidence of new forms based on PXRD were not analyzed further, and the PXRD can be found in Table S1 in the Supporting Information. One coformer listed in Table 1, NEA, displayed evidence as to the production of a new crystalline form based on the initial PXRD analysis presented in Figure 4. Initial experimental screening by LAG and SE of CEL with NEA in a 1:1 molar ratio (samples LAG8a and SE8, respectively) resulted in the formation of a solid whose PXRD displayed new peaks at positions 6.3, 7.3, 12.0, and 14.0°, along with characteristic peaks from CEL (form III) at positions 5.3, 8.9, 10.7, and 13.0°, indicating the presence of a new crystalline product. These new peaks are indicated in Figure 4 by green dots. LAG was performed again, employing an excess of the coformer (1:3 molar ratio, sample LAG8b), and this resulted in the formation of a pure new crystalline phase.

This coformer was also screened employing the two supercritical fluid techniques, GAS, employing a batch mode of operation, and SEA, employing a continuous mode of operation. The GAS method was incapable of generating the suspected new crystalline phase that was obtained from LAG. This is understood to be occurring due to the removal of the liquid coformer along with the methanol during the solid separation process once the CO_2_/solvent mixture is vented from the crystallization vessel, therefore preventing any reaction between the starting materials (i.e., crystallization of CEL with the coformer). This, however, was not the case for the SEA method. Similar to what occurred when employing the LAG method, a mixture of new peaks and characteristic CEL peaks was evident in the PXRD when a 1:1 molar ratio between the API and the coformer was employed in the feed solution. When a 1:2 molar ratio was employed in the starting solution, the powder presented a similar PXRD diffractogram to that of LAG8b (1:3 molar ratio), further providing evidence that the new product is not in a 1:1 molar ratio; rather it seems to be in a 1:2 molar ratio.

### Crystal Characterization

**Figure 5:**
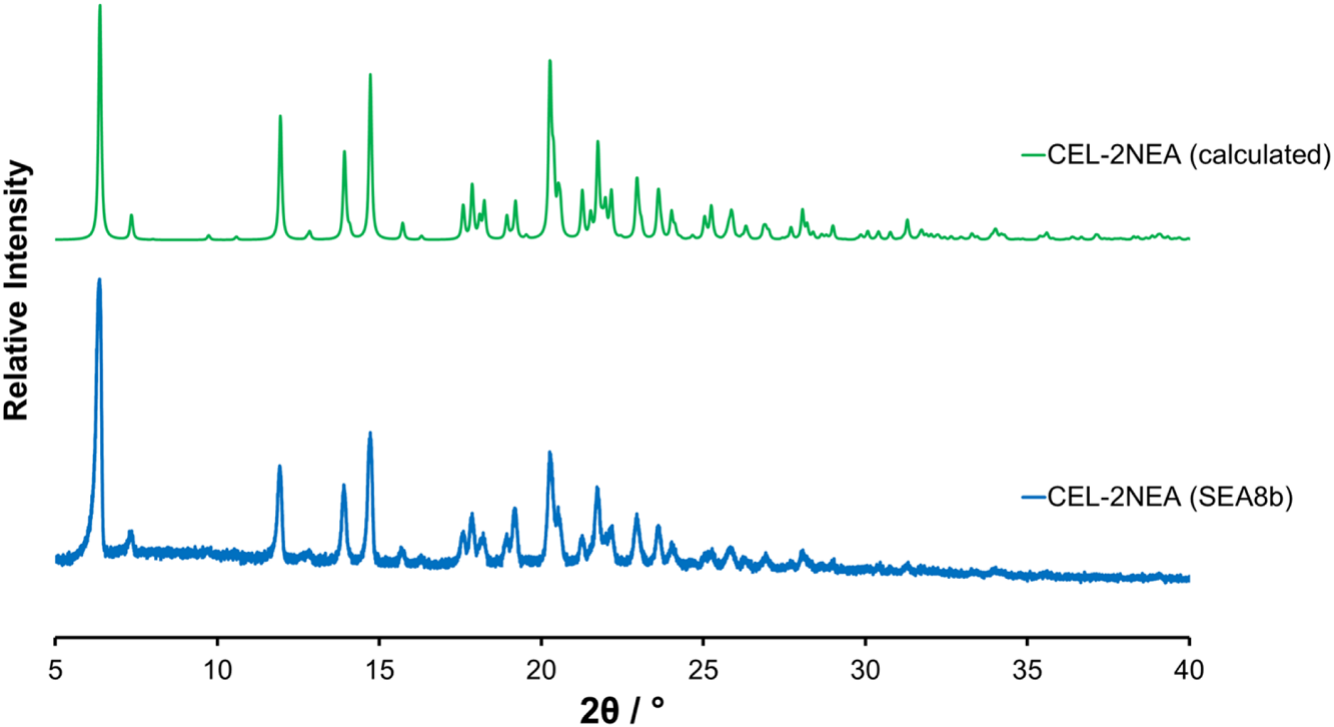
Comparison of the calculated (green) and experimental (blue) PXRD patterns for the CEL·2NEA cocrystal.

**Figure 6:**
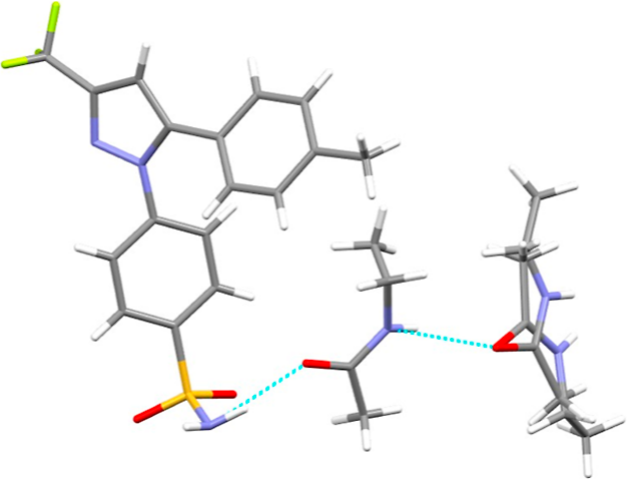
Asymmetric unit of the CEL·2NEA cocrystal with one molecule of NEA characterized by orientational disorder. Hydrogen bonds are represented by light-blue dashed lines.

The cooling of a saturated solution of CEL in NEA from 50 °C to room temperature in a Petri dish led to the formation of single crystals of the unreported CEL·2NEA cocrystal. CEL·2NEA crystallizes in a monoclinic crystal system, *P*2_1_/*c* space group, with *a* = 12.0239 Å, *b* = 27.7108 Å, *c* = 8.7569 Å, and β = 90.489° (Table 7). The calculated PXRD pattern is superimposable to the experimental results of the powder obtained by the SEA process (Figure 5). One molecule of CEL and two NEA molecule are present in the asymmetric unit, with one NEA molecule presenting orientational disorder (Figure 6). CEL is bonded to only one molecule of NEA with H-bond between the amino group of CEL and the carbonyl of NEA (N–H···O=C); the NEA molecules interact with a hydrogen bond N–H···O=C between the amide and the carbonyl group. The unit cell contains 4 molecules of CEL and 8 of NEA (*Z* = 4).

**Table 6:** Predicted Intermolecular HBPs between CEL and NEA^a^

donor	acceptor	propensity
N3_A	O3_B	0.863
N4_B	O3_B	0.813
N3_A	O1_A	0.683
N3_A	O2_A	0.683
N4_B	O1_A	0.599
N4_B	O2_A	0.599
N3_A	N1_A	0.533
N4_B	N1_A	0.443

a “A” referring to the API (CEL) and “B” referring to the coformer (NEA).

**Table 7:** Crystal Data and Details of Measurement for the CEL·2NEA Cocrystal (1:2)

	CEL·2NEA (1:2)
chemical formula	C_17_H_14_F_3_N_3_O_2_S, 2(C_4_H_9_NO)
*M*_w_, g mol^–1^	555.61
*T*/K	298
crystal system	monoclinic
space group	*P*2_1_/*c*
*a*/Å	12.0239(7)
*b*/Å	27.7108(18)
*c*/Å	8.7569(6)
α/°	90
β/°	90.489(2)
γ/°	90
*V*/Å^3^	2917.6(3)
*Z*, *Z*′	4, 1
*d*/g cm^–3^	1.265
μ/mm^–1^	0.168
measd reflns	63,722
indep reflns	5742
reflns with *I* > 2σ(*I*)	3451
*R*_int_	0.0612
*R* [*F*^2^ > 2σ(*F*^2^)]	0.0661
*wR*_2_ (*F*^2^)	0.1933

Table 6 details the predicted homomeric (API–API and coformer–coformer) and heteromeric (API–coformer) hydrogen bonds between CEL and NEA from the HBP screening. The hydrogen bond with the highest predicted propensity is a heteromeric bond between the nitrogen of the amine group of CEL and the oxygen of the carbonyl group of NEA. As seen from Figure 6 of the asymmetric unit, coupled with the details above of the realized cocrystal, this matches with the hydrogen bond observed following single-crystal analysis, between the API and coformer. In addition to this, the bond with the second highest propensity predicted was that of a homomeric hydrogen bond between two NEA molecules, between the nitrogen of the amide group and the oxygen of the carbonyl group. Interestingly, this is the realized bond observed between both NEA molecules in the asymmetric unit of this cocrystal (Figure 6). The realization of both heteromeric and homomeric hydrogen bond in the discovered cocrystal explains the low MCS value obtained for this cocrystal.

**Figure 7:**
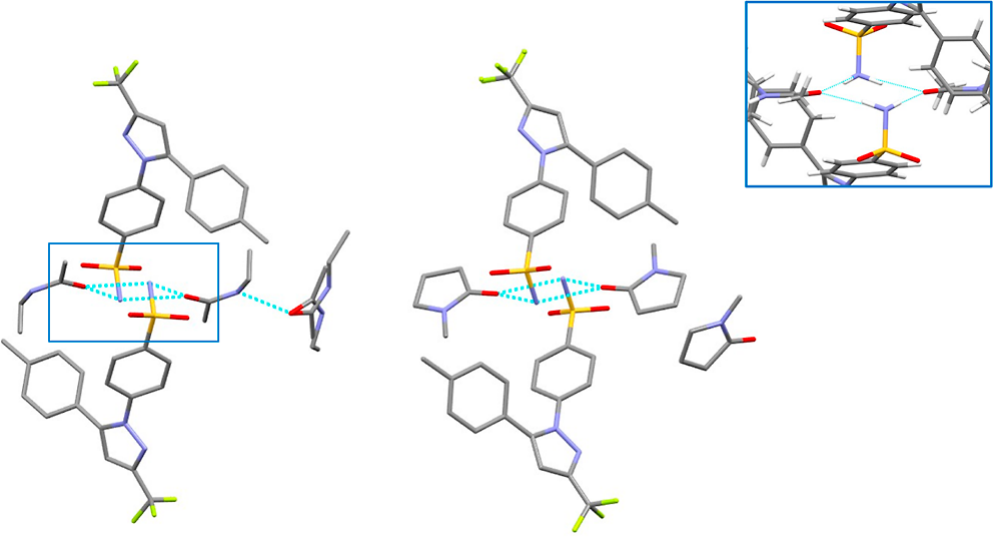
Representation of the tetramer between two CEL and two solvent molecules connected by N–H···O=C hydrogen bonds for CEL·2NEA (left) and CEL·2NMP (right); hydrogen bonds are represented in light-blue dashed lines. H atoms are removed for clarity. In the blue square, an enlargement of the R_4_^2^ (8) motif is shown.

**Figure 8:**
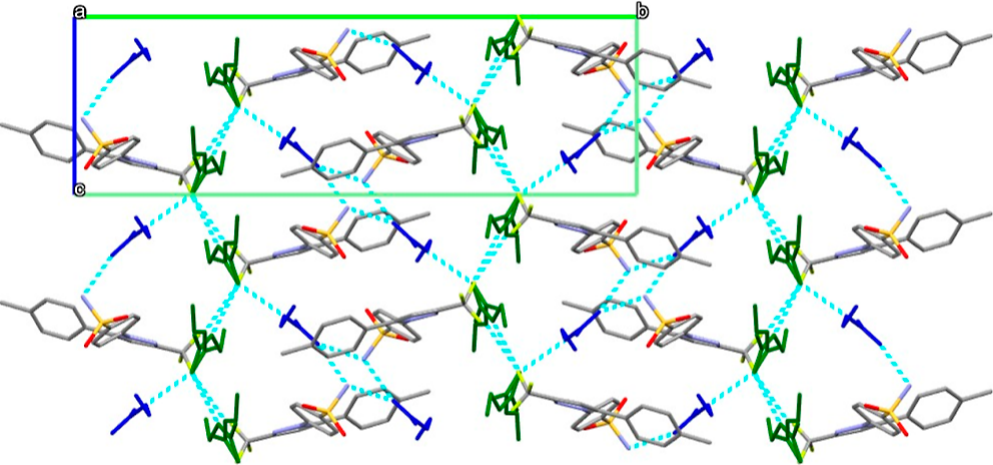
Crystal packing of the CEL·2NEA cocrystal, view along the *a*-axis; molecules of NEA not involved in the tetramer are colored in green, and the NEA molecules that form the tetramers are in blue; hydrogen bonds are represented in light-blue dashed lines, and H atoms are removed for clarity.

**Figure 9:**
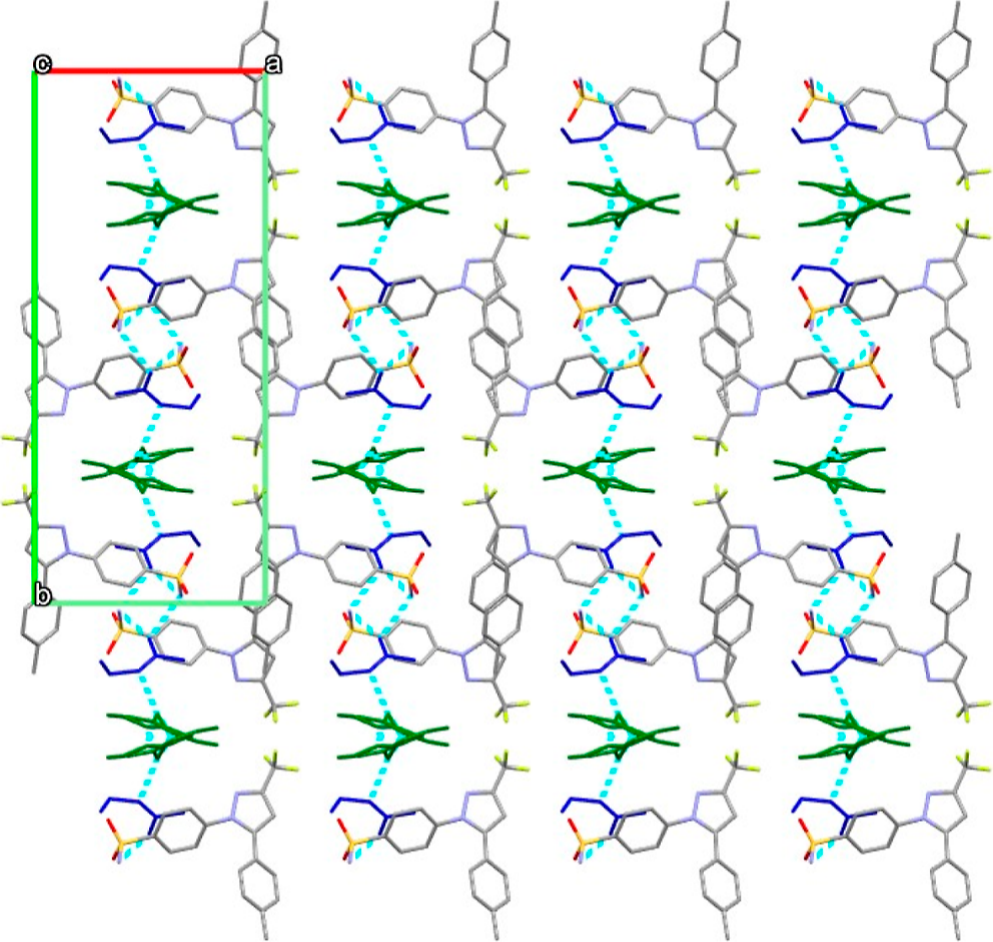
Crystal packing of the CEL·2NEA cocrystal, view along the *c*-axis; molecules of NEA not involved in the tetramer are colored in green, and the NEA molecules that form the tetramers are in blue; hydrogen bonds are represented in light-blue dashed lines, and H atoms are removed for clarity.

The CEL·2NEA cocrystal is almost isostructural to the CEL *N*-methyl-2-pyrrolidone cocrystal with 1:2 stoichiometry (CEL·2NMP) (CSD refcode WADMUN).^38^ Indeed, as observed in CEL·2NMP, the main feature of the crystal structure is a tetramer between two molecules of CEL and two of NEA sustained by strong intermolecular H-bonds (hydrogen-bonded motif R_4_^2^ (8), Figure 7). The R_4_^2^ (8) motif involves two –NH_2_ groups from CEL (the donors) and two carbonyls from the NEA molecules (the acceptors). Such supramolecular clusters packed along the *ac* crystallographic plane are held together by weak S=O···H–C bonds and dispersive forces. In contrast, along the *b* crystallographic axis shorter, the same supramolecular clusters are alternated by additional NEA molecules, which are disordered over two position and fits within pockets surrounded by the trichloromethyl groups of CEL. Ultimately, the structure appears as a layered structure along the *b* axis with ordered supramolecular CEL–NEA clusters alternated by disordered “solvent” (Figures 8 and 9). The same structural arrangement was found in CEL·2NMP; however, here, the NMP molecules of the chains are not involved in any intermolecular strong H-bonds.^38^

#### Thermal Analysis

**Figure 10:**
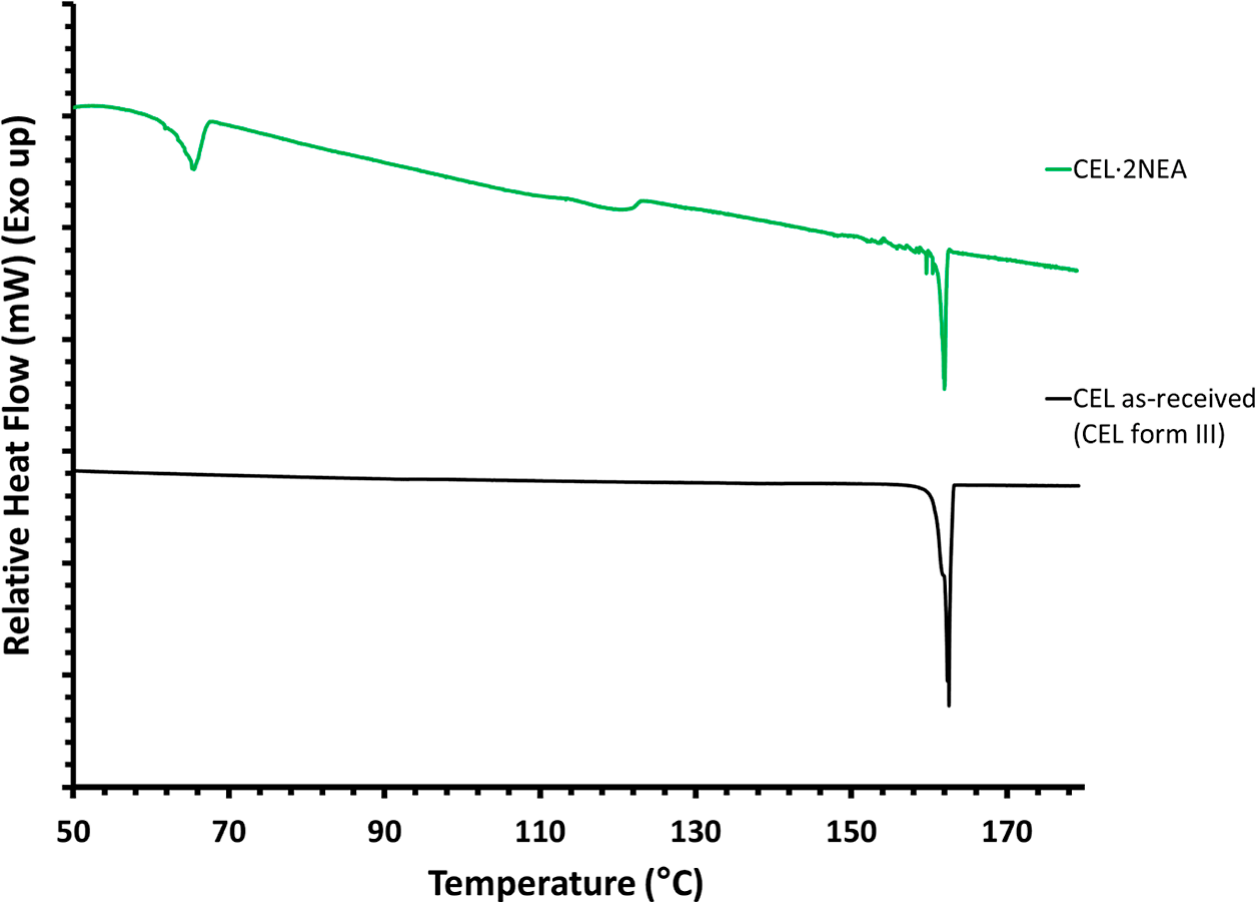
DSC analysis of as-received CEL and CEL·2NEA produced by cooling crystallization at a heating rate of 1 °C/min.

Thermal analysis was performed on CEL and the CEL·2NEA cocrystal by means of DSC, at a heating rate of 1 °C/min, with thermograms presented in Figure 10. As can be observed, CEL displays characteristic melting behavior of the stable form III, with a single endothermic peak corresponding to the melting, with an onset temperature of 161.8 °C. The CEL·2NEA cocrystal, however, displays two endothermic peaks, with onsets of 63.6 and 161.2 °C. The first endothermic peak corresponds to desolvation and removal of NEA from the crystal lattice, while the second endothermic peak corresponds to the melting of the crystalline form, closely matching the reported melting ranges for several CEL polymorphs, namely, I, II, and III. A downward slope is also noted in the thermogram for CEL·2NEA. This is due to the partial dissolution of CEL in NEA following from desolvation occurring at a temperature significantly lower than the boiling point of NEA.^38^ A small exothermic event can also be observed in the CEL·2NEA thermogram at approximately 122 °C, which likely corresponds to the recrystallization of the desolvated CEL into a stable crystalline polymorphic form.

**Figure 11:**
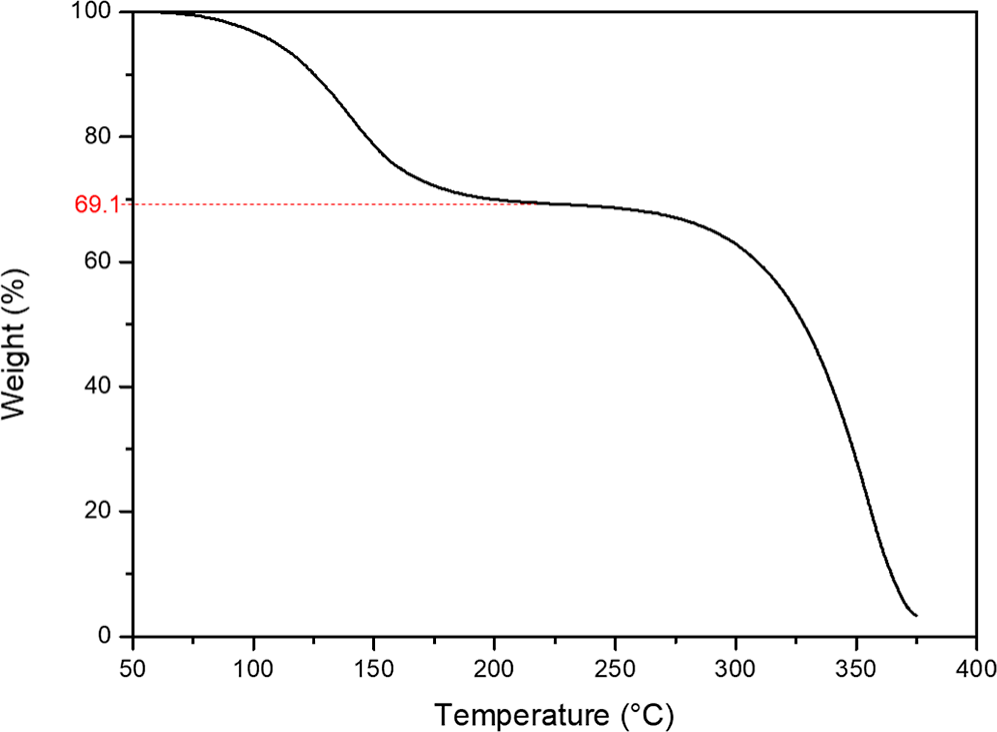
TGA of the new CEL·2NEA cocrystal at a heating rate of 10 °C/min.

Analysis by means of TGA was also performed and is presented in Figure 11, to determine the weight loss upon heating. The weight remaining from the desolvation event is marked in red at 69.1% weight remaining. This corresponds to a molar ratio of CEL·2NEA of 1:1.96, matching closely with the ratio of 1:2 as determined by structural analysis by SC-XRD.

#### VT-PXRD

**Figure 12:**
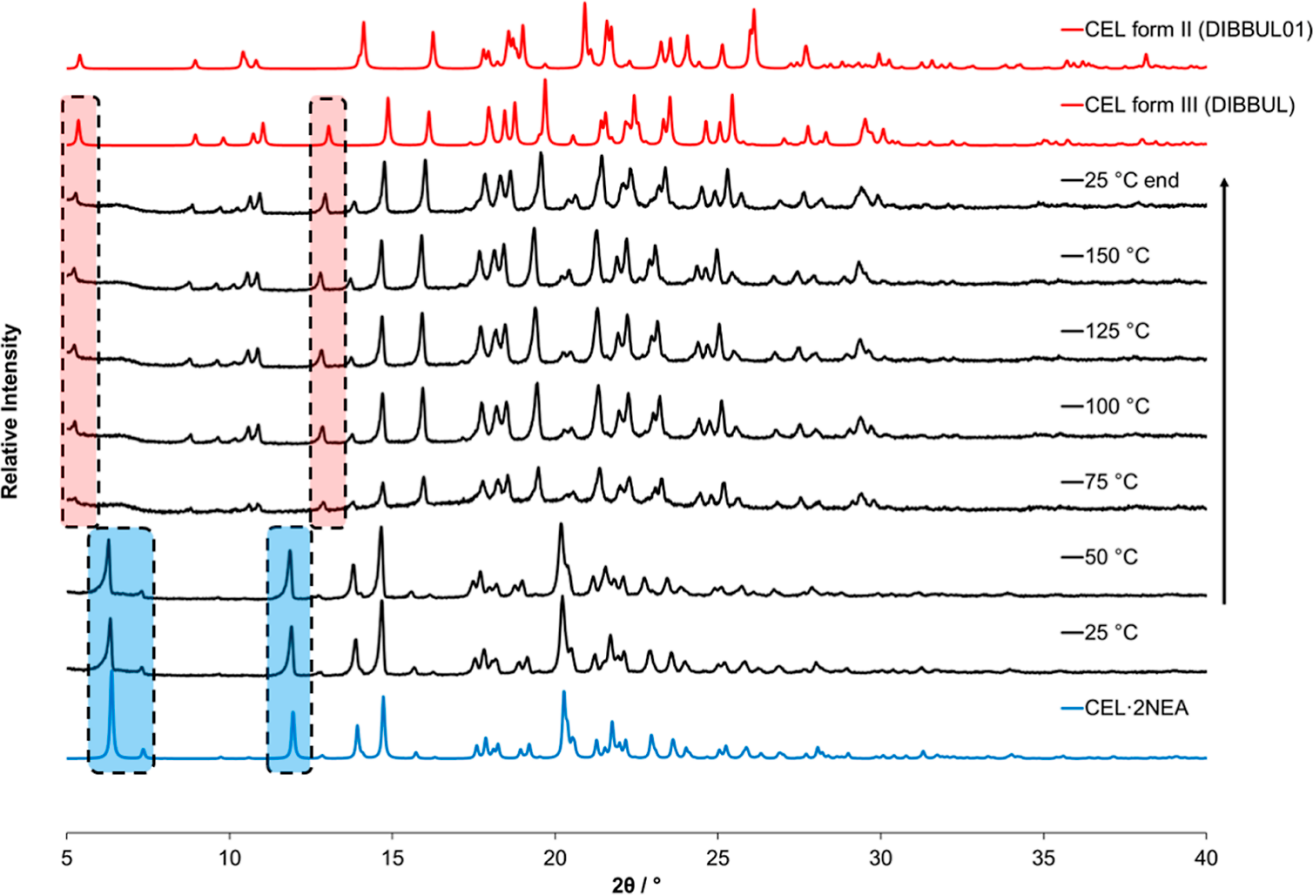
Diffractograms obtained from VT-PXRD analysis of CEL·2NEA produced by SEA, at a heating rate of 10 °C/min from 25 to 150 °C and a cooling rate of 10 °C/min from 150 to 25 °C.

As the melting temperatures of the various polymorphic forms of CEL are only a few degrees in difference, it is difficult to identify the desolvated form of CEL from the DSC analysis in Figure 10 by the melting temperature. For this reason, VT-PXRD was performed, the results of which are presented in Figure 12 below. The VT-PXRD analysis reveals that the desolvation process of CEL·2NEA occurs between 50 and 75 °C (in agreement with the DSC and TGA analyses). During this temperature range, the cocrystal transforms to CEL form III. One small residual peak is also present at 14.1°, which may correspond with form II or CEL·2NEA.

Samples were also placed in an oven at 65 °C and analyzed periodically by PXRD, the results of which are provided in Figure S2 in the Supporting Information.

The thermal analyses and the VT-PXRD indicate that the desolvation process of CEL·2NEA consists of the loss at the same temperature of all NEA molecules, both those involved in the tetramers and those arranged in the infinite chains. This results in the direct formation of CEL form III from the CEL·2NEA without passing through a hypothetical form such as CEL mono-NEA, as observed in the case of CEL·2NMP, where the desolvation process leads to the mono-NMP. The reason is that in CEL·2NMP, the NMP molecules in the chains are not tightly bound through strong intermolecular interactions allowing them to escape from the crystal structure without affecting the CEL and NMP molecules arranged in the tetramers.^38^ In CEL·2NEA, however, all NEA molecules in the crystal structure are connected through strong intermolecular H-bonds; therefore, when the desolvation process starts, the crystal structure collapses, and all NEA molecules are released at the same time; this hypothesis could also be supported by the formation of a partially amorphous phase at 75 °C following the desolvation process (Figure 12).

#### Crystal Habit Analysis

**Figure 13:**
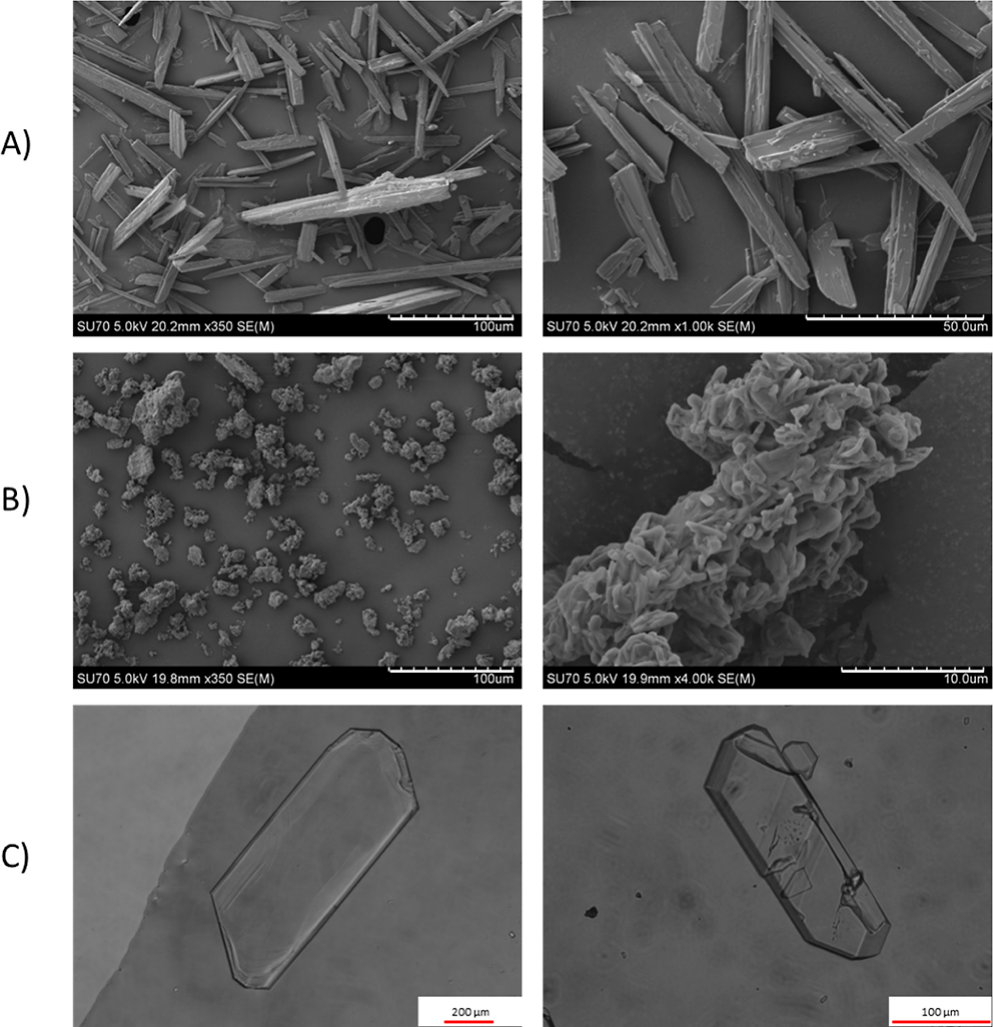
A) SEM images of as-received CEL, (B) SEM images of CEL·2NEA produced by the SEA process (SEA8b), and (C) optical microscopy images for CEL·2NEA single crystals produced from cooling crystallization.

The SEM and optical microscopy images of the samples produced in this work are presented in Figure 13, along with SEM images of the as-received CEL (form III). The as-received CEL presents an elongated plate-like habit that could also be described as needlelike, with particle lengths ranging from 50 to 150 μm. The single crystals of CEL·2NEA produced by cooling crystallization from a saturated solution of CEL in NEA display a plate-like habit of a relatively large size, with some particles displaying a length longer than 1.2 mm. SEM images of the SEA-produced CEL·2NEA sample displayed many small agglomerates. These agglomerates appear to be composed of smaller granular particles.

#### Fourier Transform Infrared Spectroscopy

**Figure 14:**
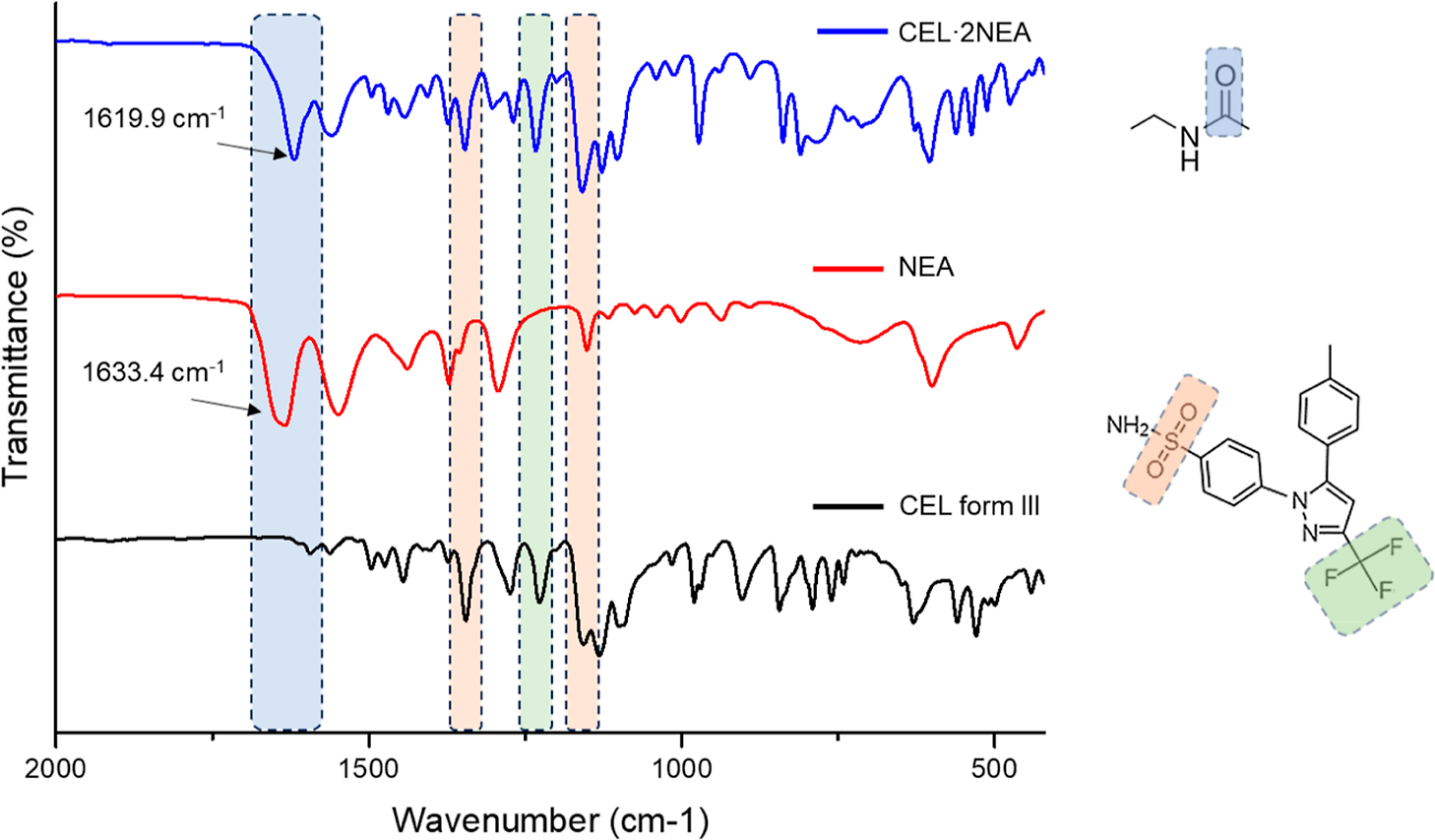
FTIR spectroscopy spectra of CEL form III (as-received), NEA, and CEL·2NEA produced by SEA.

FTIR was used to further investigate the structure of the new cocrystal (CEL·2NEA) and its hydrogen bonding network. The IR spectra of as-received CEL, NEA, and the CEL·2NEA cocrystal are detailed, and the most relevant bands are highlighted in Figure 14. The CEL·2NEA cocrystal and CEL have bands at 1158 cm^–1^ (O=S=O asymmetric stretching), 1233 cm^–1^ (CF_3_ stretching), and 1346 cm^–1^ (O=S=O symmetric stretching). A single band for NEA is present at 1634 cm^–1^ corresponding to the C=O stretching, which is not present in the spectra for CEL. This is, however, present in the cocrystal, but at a lower frequency. Lowering of frequencies for the C=O stretching from 1635 cm^–1^ in NEA to 1619 cm^–1^ in the cocrystal is due to the strong hydrogen bonds that all of the NEA molecules form: in the tetramer with the –NH_2_ groups of CEL and among themselves in the chains with the amide group. This results in a weakening of the double bond. In contrast to the CEL·2NEA cocrystal which presents a single peak for C=O stretching, the CEL·2NMP cocrystal^38^ presents two peaks for C=O stretching corresponding to the H-bonded and non-H-bonded C=O group of the two NMP molecules. The presence of this single band for CEL·2NEA corresponding to C=O stretching further confirms the structure we report here, due to both NEA molecules being involved in H-bonding. Thus, the FTIR analysis provided here corroborates the crystallographic information obtained from SC-XRD.

#### Dissolution Study

**Figure 15:**
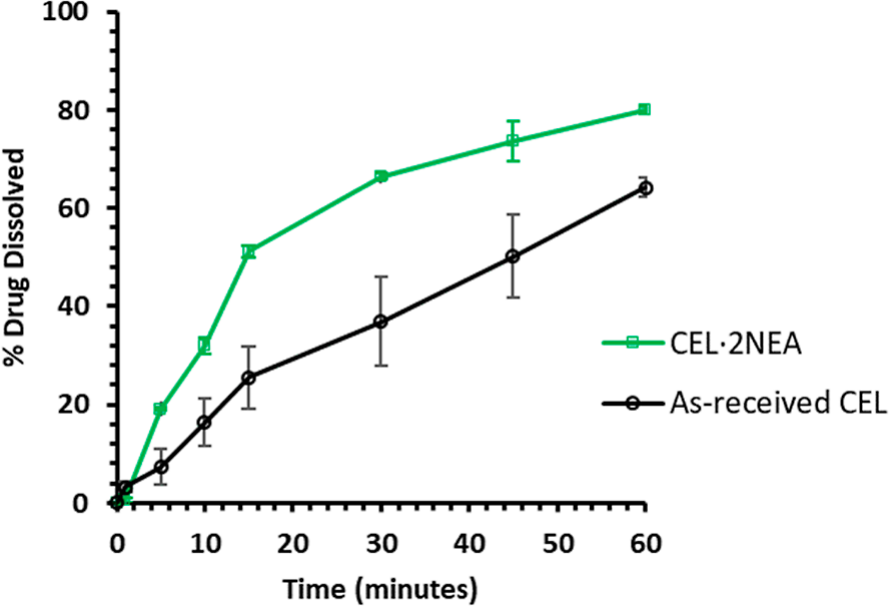
Dissolution profiles of the as-received CEL and CEL·2NEA cocrystal produced by cooling crystallization. Dissolution conditions: 200 mL of 0.25% w/v SDS in deionized water, 37 ± 1 °C, 300 rpm, [CEL]_max_ = 7 mg/L.

Dissolution profiles of as-received CEL and CEL·2NEA (produced by cooling crystallization) are presented in Figure 15. SDS was included in the dissolution media (0.25% w/v) due to the excessively poor solubility of CEL making detection at early time points in high-performance liquid chromatography (HPLC) difficult. Both samples examined here displayed 100% dissolution at 24 h, and Figure 15 displays the dissolution profile generated during the first 60 min. A more rapid dissolution is seen for CEL·2NEA in the first 15 min with more than a twofold improvement in the dissolution rate in comparison to that of CEL. The dissolution curve for as-received CEL, however, is significantly slower, achieving only 25% drug dissolved in the first 15 min. The decrease of particle size to improve dissolution rate is only significant once below 1 μm.^39^ The influence of particle size on dissolution here in Figure 15 can therefore be negated as all particles are larger micron-sized particles. As shown in the SEM images in Figure 13, the particle size for as-received CEL is 50–150 μm, and approximately 1 mm for CEL·2NEA. It is noteworthy that a previous reported cocrystal of CEL with the coformer nicotinamide also displayed an improvement in the dissolution rate in comparison to the CEL form III, with and without the inclusion of SDS.^25^

## Conclusions

Despite the therapeutic benefits of API CEL, its clinical applications are extremely limited by its poor aqueous solubility. In this work, a large and comprehensive computational screening study was conducted to aid in the discovery of a new multicomponent solid form of CEL, to reduce the labor associated with experimental screening of a large number of coformer candidates. The combination of virtual and experimental screening has successfully led to the discovery of a new cocrystal form of CEL with the coformer NEA. While methods such as LAG are valuable in rapid experimental screening, they introduce difficulties regarding the control of the molar ratio during the use of liquid coformers. Similarly, while GAS is a more rapid screening technique than SEA, difficulties are also encountered when using liquid coformers, as they can often be removed from the reaction vessel, preventing cocrystallization from occurring. While SEA may be a lengthy process in comparison to LAG, it may be preferred over methods such as SE that can be lengthened when incorporating solvents with high boiling points. The production of single crystals and subsequent crystal structure determination by SC-XRD analysis, coupled with TGA data, confirmed the molar ratio of CEL/NEA to be 1:2. The structure is considered quasi-isostructural to a previously reported cocrystal of CEL with *N*-methyl-2-pyrrolidone, while exhibiting different thermal behavior with respect to the release of solvent molecules upon heating. VT-PXRD and DSC analysis conform that complete desolvation occurs at approximately 65 °C, at which point VT-PXRD highlights the presence of CEL form III, as well as residual quantities of CEL form II. The newly reported cocrystal displays a twofold improvement in the dissolution rate at only 15 min in comparison to the commercially available form III of CEL.

## Supporting Information Available

Virtual screening results (XLSX)
Summary of all data in relation to the virtual screening (CIF)
Chemical structures of all coformers investigated experimentally; PXRD patterns of experimental screenings of coformers which did not present any evidence as to the presence of a new solid form of CEL; summary of coformers which were virtually screened; PXRD patterns for the CEL·2NEA (celecoxib-2-*N*-ethylacetamide) sample produced by SEA held at 65 °C and analyzed after 0, 6, 24, and 48 h to determine the time required for complete desolvation and transformation into a stable crystalline form of CEL; and all peaks corresponding to the CEL·2NEA solvate form have disappeared and only peaks corresponding to CEL form III remained after 48 h (PDF)

## Supplementary Material

ao4c03188_si_001.xlsx

ao4c03188_si_002.cif
