# Supplementary material for: Screening, Synthesis, and Characterization of a More Rapidly Dissolving Celecoxib Crystal Form
Source: ACS Omega. 2024 Jun 27;9(27):29710–22. doi: 10.1021/acsomega.4c03188 (PMC11238285; doi:10.1021/acsomega.4c03188)
Supplement: Supplementary file 3 — ao4c03188_si_003.pdf [file ao4c03188_si_003.pdf]

# Supplementary Information

# Screening, synthesis, and characterization of a more rapidly dissolving celecoxib crystal form

Aaron O'Sullivan[1,2], Enrico Spoletti[2], Steven A. Ross[4], Matteo Lusi[2], Dennis Douroumis[3], Kevin M. Ryan[1,2], Luis Padrela[1,2]*

1. SSPC Research Centre, University of Limerick, Limerick, Ireland
2. Department of Chemical Sciences, Bernal Institute, University of Limerick, Limerick V94 T9PX, Ireland
3. CIPERinitio Centre for Innovation and Process Engineering Research, University of Greenwich, Chatham, Maritime Kent ME4 4TB, United Kingdom
4. Custom Pharma Services, Brighton and Hove, East Sussex, UK

* Corresponding author: luis.padrela@ul.ie

**Content:**

Figure S1. Chemical structures of all coformers investigated experimentally.

Table S1. PXRD patterns of experimental screenings of coformers which did not present any evidence as to the presence of a new solid form of celecoxib.

Table S2. Summary of coformers which were virtually screened.

Figure S2. PXRD patterns for the CEL·2NEA (celecoxib.2 N-ethylacetamide) sample produced by SEA (supercritical enhanced atomization) held at 65°C and analysed after 0, 6, 24 and 48 hours to determine the time required for complete desolvation and transformation into a stable crystalline form of CEL (celecoxib). After 48 hours, all peaks corresponding to the CEL·2NEA solvate form have disappeared and only peaks corresponding to CEL form III remained.

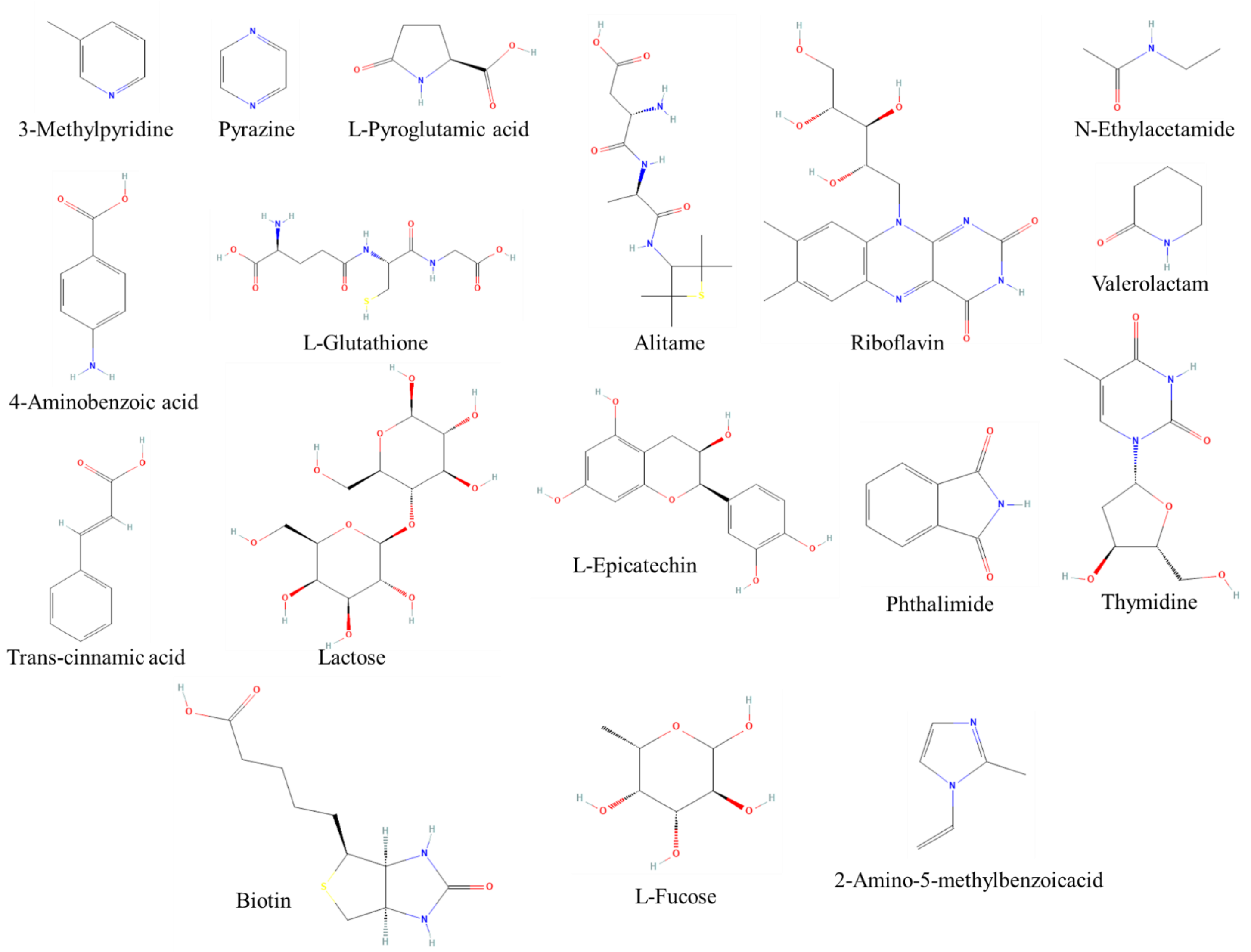


***Figure S1.*** *Chemical structures of all coformers investigated experimentally.*

***Table S1.*** *Powder X-ray diffraction (PXRD) patterns of experimental screenings of coformers which did not present any evidence as to the presence of a new solid form of celecoxib.*

| Ranking | Coformer | PXRD |
|---|---|---|
| 1. | 3-Methylpyridine | 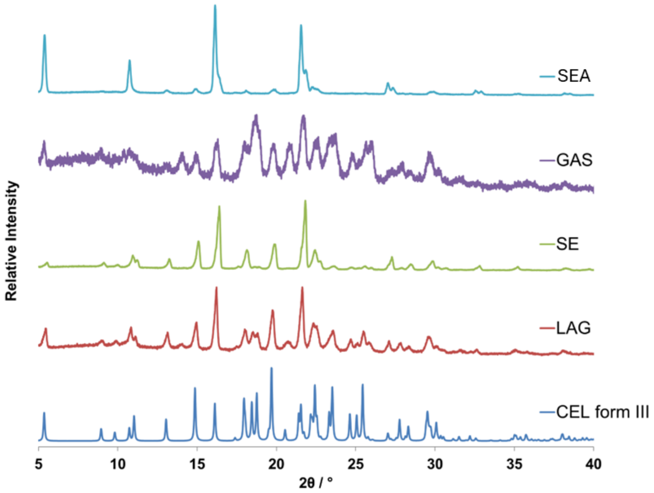 |
| 2. | Pyrazine | 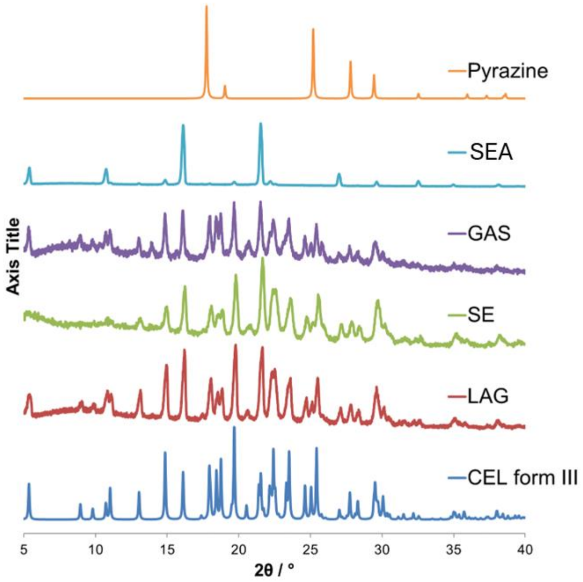 |

| | | |
|---|---|---|
| 3. | L-Pyroglutamic acid | 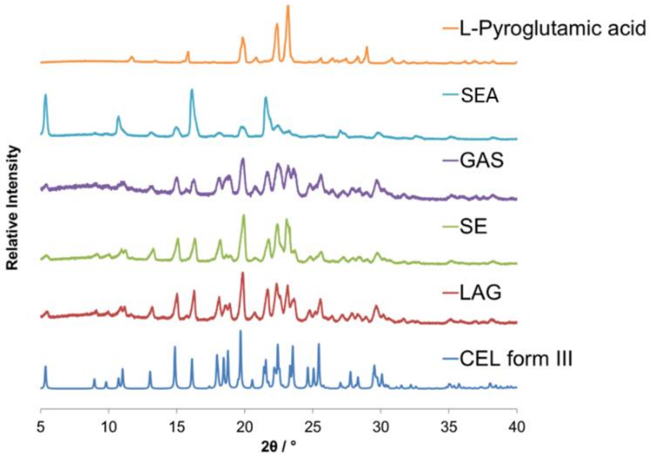 |
| 4. | Riboflavin | 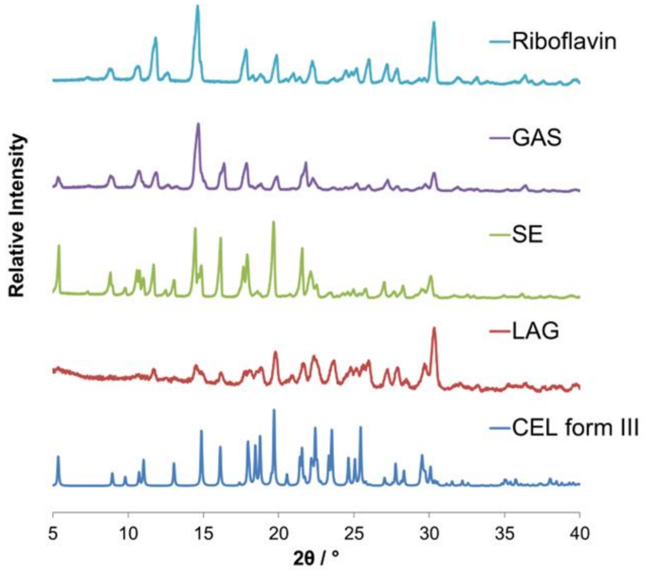 |
| 5. | Alitame | 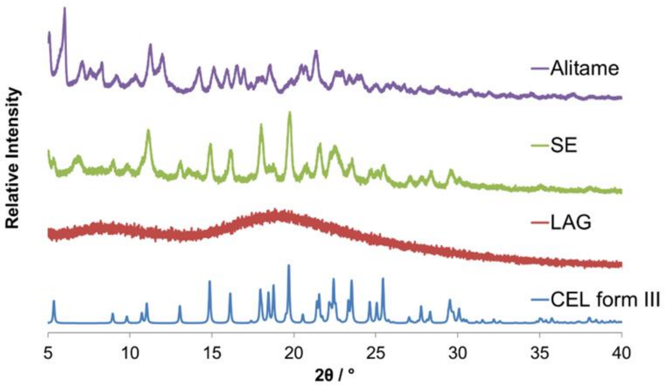 |

| | | |
|---|---|---|
| 6. | 4-Aminobenzoic acid | 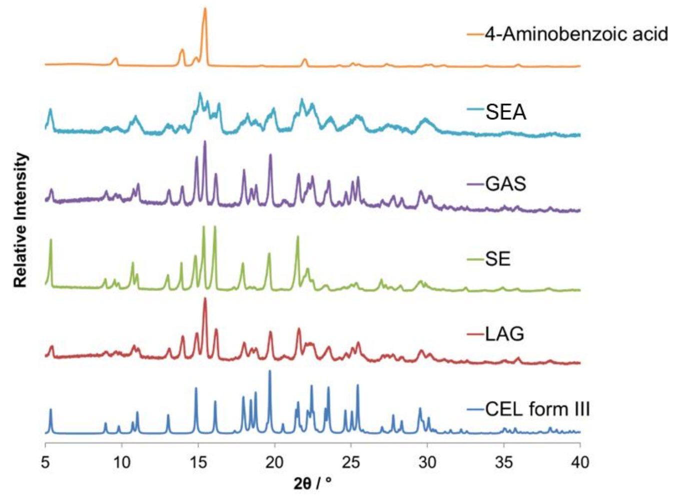 |
| 7. | L-Glutathione | 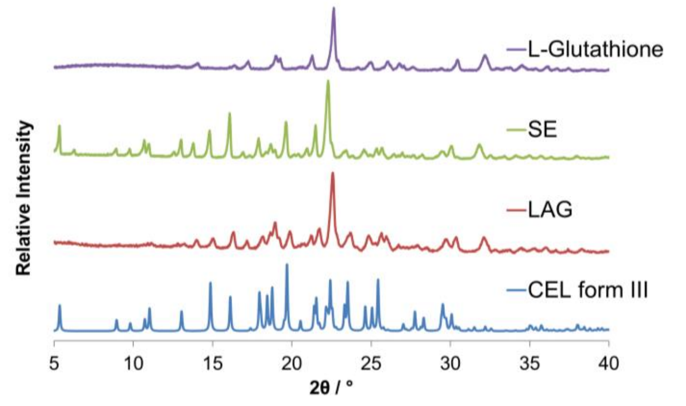 |
| 9. | Trans-cinnamic acid | 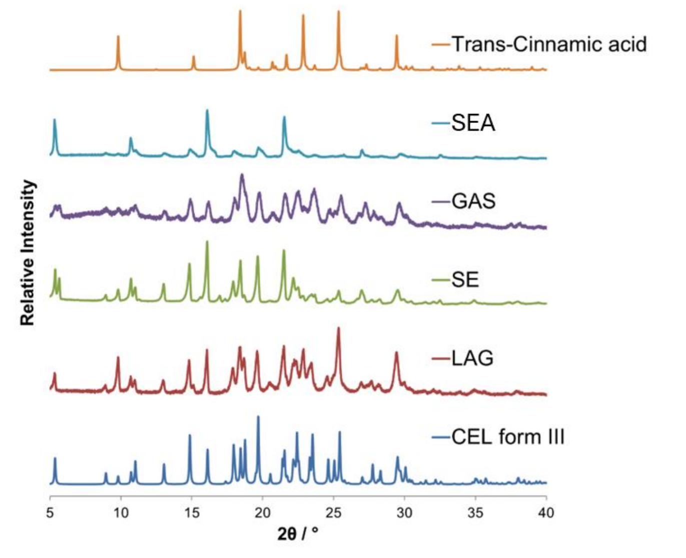 |

| | | |
|---|---|---|
| 10. | Valerolactam | 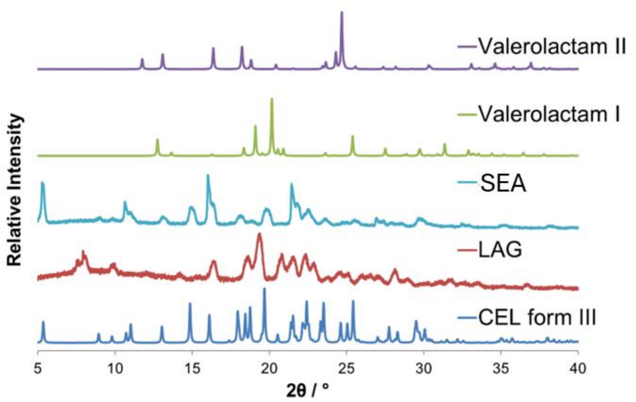  |
| 11. | Lactose | 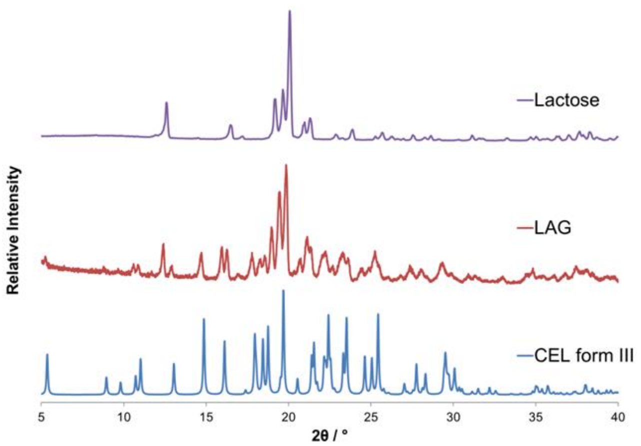  |
| 12. | L-Epicatechin | 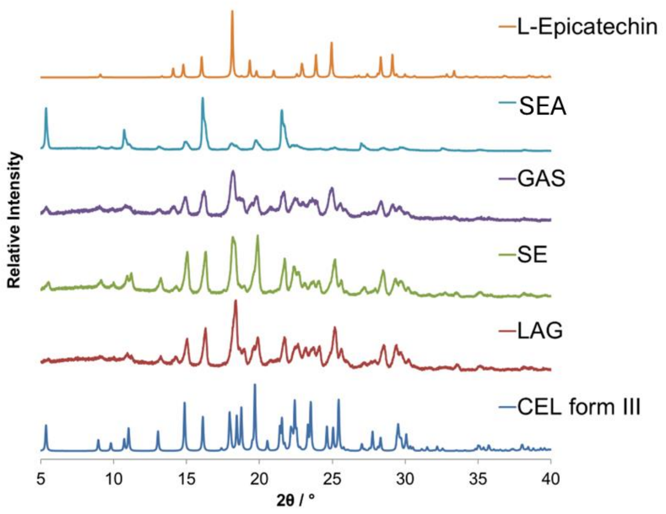  |

| | | |
|---|---|---|
| 13. | Thymidine | 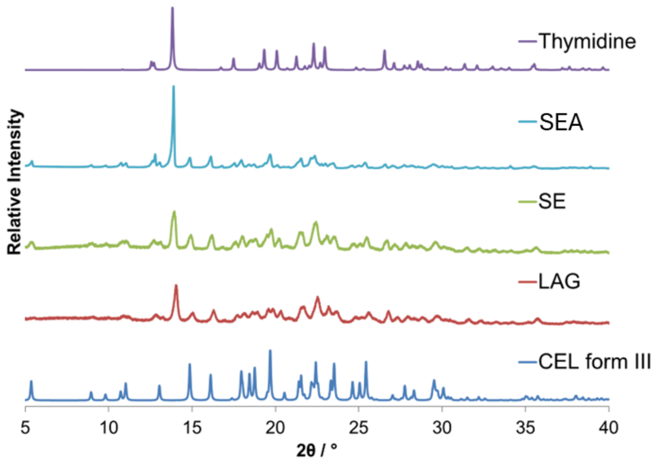  |
| 14. | Biotin | 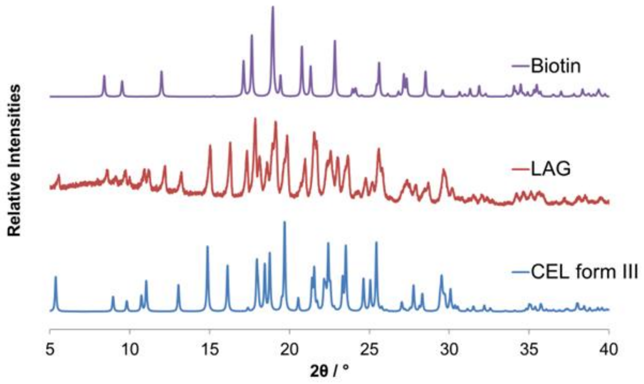  |
| 15. | Phthalamide | 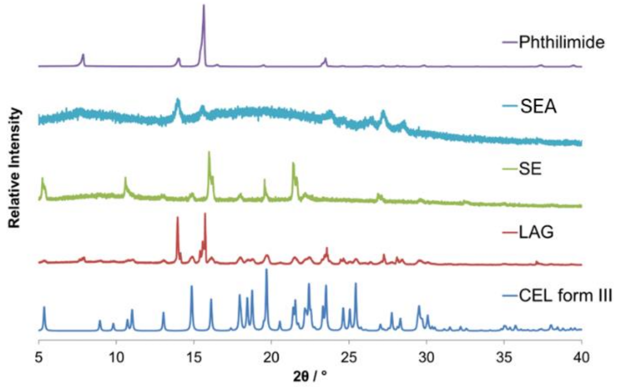  |

| | | |
|---|---|---|
| 16. | L-Fucose | 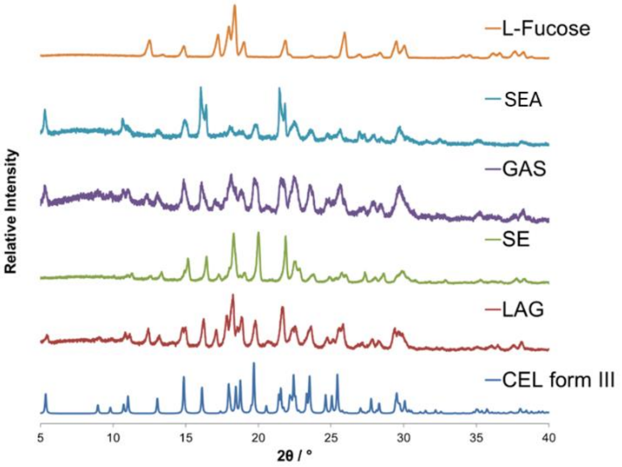  |
| 17. | 2-Amino-5-Methyl Benzoic acid | 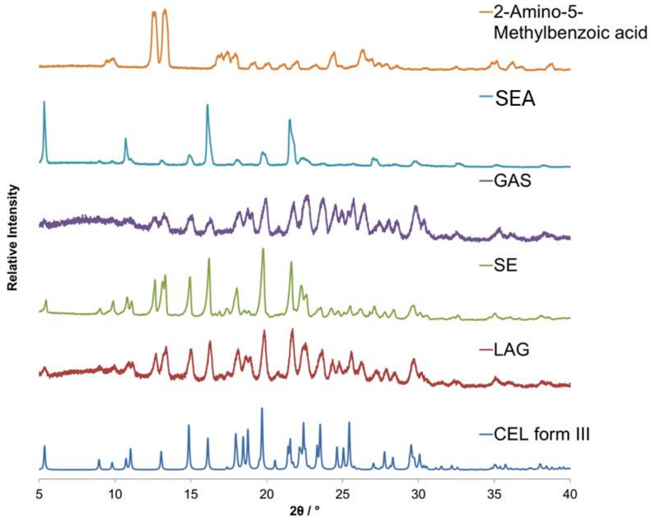  |

CEL, Celecoxib; LAG, liquid-assisted grinding; SE, solvent evaporation; GAS, gas antisolvent crystallization; SEA, supercritical enhanced atomization.

***Table S2.*** *Summary of coformers which were virtually screened.*

| **Coformer** |
|---|
| (-)-camphorsulfonic_acid |
| (+)-camphoric_acid |
| 1-hydroxyethylidene-1,1-diphosphonic_acid |
| 1-Methylamino-D-1-deoxy-glucitol |
| 2-(3,4-Dihydroxyphenyl)chromane-3,5,7-triol methanol solvate |
| 2-(4'-Thiazolyl)benzimidazole |
| 2,6-Di-t-butyl-4-methylphenol |
| 2-amino-5-methylbenzoic_acid |
| 3-Ethoxy-4-hydroxybenzaldehyde |
| 3-methylpyridine |
| 4-acetamidobenzoic_acid |
| 4-aminobenzoic_acid |
| 4-hydroxybenzoic_acid |
| acesulfame |
| acetic_acid |
| acetophenone_oxime |
| acetylenedicarboxylic_acid |
| adipic_acid |
| alitame |
| apigenin |
| azelaic_acid |
| benzoic_acid |
| biotin |
| caprolactam |
| capsaicin |
| cholic_acid |
| citric_acid |
| D-alanine |
| D-glucuronic_acid |
| DL-Mandelic acid |
| D-pantothenol |
| EDTA |
| ethylparaben |
| folic_acid |
| fumaric_acid |
| Genisteine |
| gentisic_acid |
| glutaric_acid |
| glycine |
| glycolic_acid |
| hesperetin |
| hippuric_acid |
| hydrocinnamic_acid |

| |
|---|
| imidazole |
| isonicotinamide |
| ketoglutaric_acid |
| lactobionic_acid |
| lactose |
| L-arginine |
| L-aspartic_acid |
| L-aspartic_acid_z |
| L-Fucose |
| L-glutamic_acid |
| L-glutamic_acid_z |
| L-glutamine |
| L-glutathione |
| L-Histidine |
| L-lactic_acid |
| L-leucine |
| L-mandelic_acid |
| L-methionine |
| L-phenylalanine |
| L-proline |
| L-Pyroglutamic  acid |
| L-serine |
| L-tartaric_acid |
| L-tryptophan |
| L-tyrosine |
| M00079 |
| maleic_acid |
| malic_acid |
| malonic_acid |
| maltitol |
| mannitol |
| methanesulfonic_acid |
| methylparaben |
| monobutyrin |
| N-ethylacetamide |
| nicotinamide |
| oxalic_acid |
| P-aminobenzoic  acid |
| pamoic_acid |
| phthalamide |
| P-Hydroxy-trans-cinnamic  acid |
| pimelic_acid |
| piperazine |
| propylparaben |
| pyrazine |
| riboflavin |

| |
|---|
| saccharin |
| sorbic_acid |
| suberic_acid |
| succinic_acid |
| t-butylamine |
| t-butylhydroxyanisole |
| theophylline |
| thymidine |
| Trans-cinnamic acid |
| triphenylacetic_acid |
| tris(Hydroxymethyl)aminomethane |
| urea |
| valerolactam |
| xanthine |

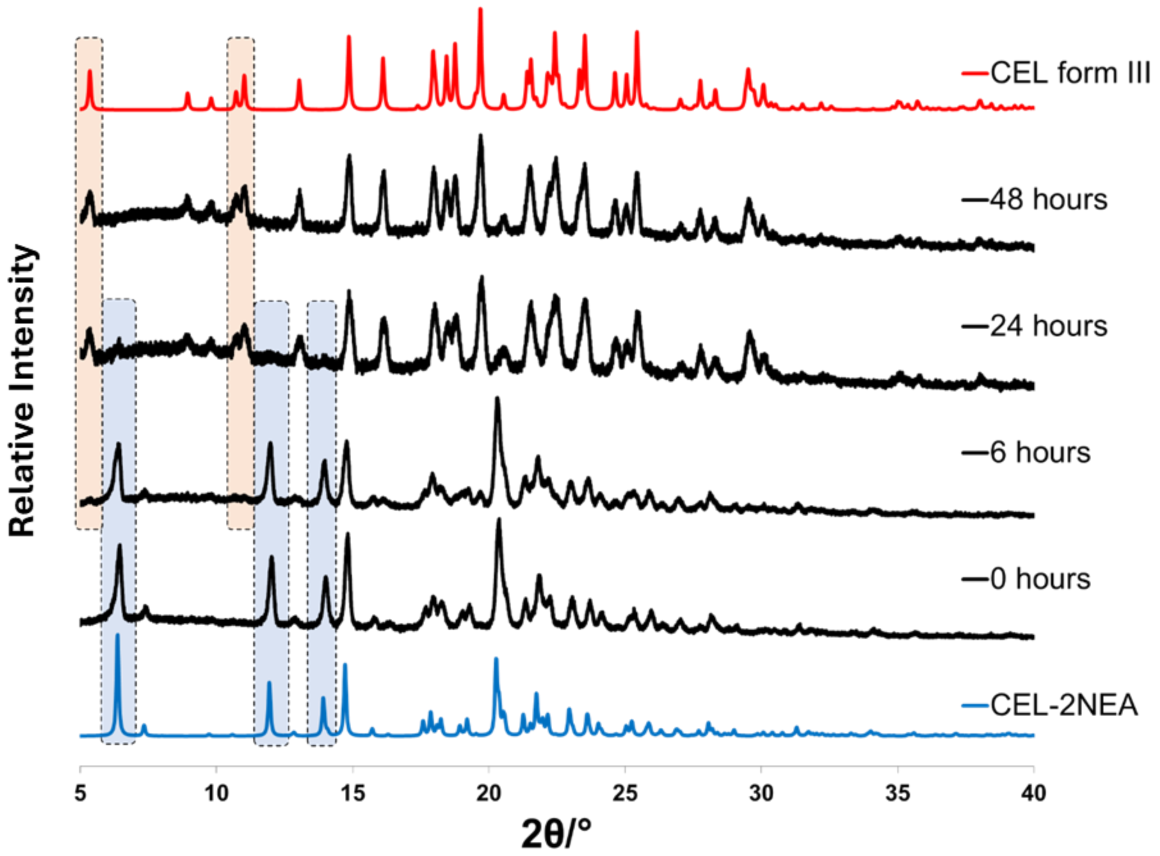


***Figure S2.*** *Powder X-ray diffraction (PXRD) patterns for the CEL·2NEA (celecoxib.2 N-ethylacetamide) sample produced by SEA (supercritical enhanced atomization) held at 65°C and analysed after 0, 6, 24 and 48 hours to determine the time required for complete desolvation and transformation into a stable crystalline form of CEL (celecoxib). After 48 hours, all peaks corresponding to the CEL·2NEA solvate form have disappeared and only peaks corresponding to CEL form III remained.*
